# Magnetic Nanoparticles Fishing for Biomarkers in Artificial Saliva

**DOI:** 10.3390/molecules25173968

**Published:** 2020-08-31

**Authors:** Arpita Saha, Hamdi Ben Halima, Abhishek Saini, Juan Gallardo-Gonzalez, Nadia Zine, Clara Viñas, Abdelhamid Elaissari, Abdelhamid Errachid, Francesc Teixidor

**Affiliations:** 1 Inorganic Materials Laboratory, Institut de Ciencia de Materials de Barcelona (ICMAB-CSIC), Campus de la UAB, 08193 Bellaterra, Spain; arpitasaha666@gmail.com (A.S.); ab76779@gmail.com (A.S.); clara@icmab.es (C.V.); 2 Micro & Nanobiotechnology Laboratory, Université de Lyon, CNRS, University Claude Bernard Lyon 1, Institut des Sciences Analytiques, UMR 5280, 5 rue de la Doua, F-69100 Villeurbanne, France; ben.halima.hamdi@hotmail.com (H.B.H.); juagalgon@gmail.com (J.G.-G.); Nadia.zine@univ-lyon1.fr (N.Z.); 3 LAGEPP-UMR 5007, CNRS, University Claude Bernard Lyon-1, University of Lyon, F-69622 Lyon, France; abdelhamid.elaissari@univ-lyon1.fr

**Keywords:** immobilization of antibodies, IL-10, magnetic nanoparticles, pre-concentration of antigens, saliva matrix, TNF-α

## Abstract

Magnetic nanoparticles (MNPs) were synthesized using the colloidal co-precipitation method and further coated with silica using the Stöber process. These were functionalized with carboxylic and amine functionalities for further covalent immobilization of antibodies on these MNPs. The procedure for covalent immobilization of antibodies on MNPs was developed using 1-ethyl-3-(dimethylaminopropyl)carbodiimide (EDC) and *N*-hydroxysuccinimide (NHS). The evaluation of the efficiency of the coupling reaction was carried out by UV-vis spectrophotometry. The developed antibodies coupled to MNPs were tested for the pre-concentration of two biomarkers tumor necrosis factor alpha (TNF-α) and Interleukin-10 (IL-10). Both biomarkers were assessed in the matrix based on phosphate-buffered saline solution (PBS) and artificial saliva (AS) to carry out the demonstration of the format assay. Supernatants were used to determine the number of free biomarkers for both studies. Reduction of the nonspecific saliva protein adsorption on the surface of the complex antibodies-MNPs to levels low enough to allow the detection of biomarkers in complex media has been achieved.

## 1. Introduction

Superparamagnetic iron oxide nanoparticles (SPIONs) are of great importance when grafted with biomarkers for applications in modern biological and biotechnology areas. The surface-modified magnetic nanoparticles (MNPs) can be used both in in-vitro and in-vivo systems effectively. The size of the MNPs needs to be controlled and innovative functionalization techniques need to be utilized for the effective implementation of these modified MNPs in medical applications [1,2,3,4,5,6].

The small size of MNPs (a few nanometers) is essential for them to be able to interact, bind, or penetrate biological entities. This is because in the nano range, their dimensions are comparable to those of proteins, cells, or viruses, and this facilitates their movement through biological structures [7]. These MNPs are highly attractive for use in biomedical applications such as magnetic resonance imaging (MRI), targeted drug delivery, and treatment of hyperthermia. These are due to certain unique properties that arise due to a combination of their small dimension, enhanced sensing, and nanoscale-dependent magnetism, as well as physiological properties [8]. MNPs are also very commonly used these days for in-vivo applications as drug carriers in a magnetic ‘tag drag-release’ process, termed as targeted drug delivery. The MNPs are usually loaded with special drug molecules or chemotherapy agents, which are directly vectorized to tumor cells by targeting ligands on their surfaces, or by the application of an external magnetic field. In recent years, substantial interest has been focused on multifunctional MNPs in which diagnostic (MRI) and therapeutic (hyperthermia treatment and drug delivery) capabilities are combined [9,10,11,12].

The successful design of MNPs for biological applications needs a careful selection of magnetic core and surface coating material, where the former mainly determines the MNPs’ heating and sensing capabilities with regards to application efficiency and the latter specifies the interaction of these MNPs with a physiological environment. To enable the direct use of MNPs in biomedical applications, the MNPs should be further functionalized by conjugating them with functional groups. The surface coating provides a suitable base for the attachment of these functional groups on MNPs. These groups such as antibodies, peptides, polysaccharides, etc., permit specific recognition of cell types and direct the MNPs to a specific tissue or cell type by binding to a cell surface receptor. The silica coating facilitates the functionalization of the surface of the MNPs with either amine groups or carboxylic groups, which help in bonding with biological entities for medical applications. Also, the silica coating does not affect the magnetic property of the Fe_3_O_4_ core in a substantial way. Among the different types of MNPs, iron oxides (magnetite (Fe_3_O_4_), maghemite (γ-Fe_2_O_3_), and hematite (α-Fe_2_O_3_)) are by far the most commonly employed ones for in-vivo applications since iron is physiologically well tolerated. Silica is inorganic but bio-friendly and it is known for its chemical stability and ease of formation. The biggest advantage of having a surface enriched in silica is the presence of silanol groups, which can easily react with coupling agents, providing strong attachment of surface ligands on MNPs [13]. There are several successful methods available for the formation of silica coating, amongst which the most commonly used is the Stöber method. Here, a hydrolysis reaction of tetraethyl orthosilicate (TEOS) is governed in alcohol media under the catalytic action of ammonia [14]. In this paper, a variation of this method is applied to produce more homogeneous coatings of silica.

The coupling strategy used for linking the functionalizing agent to the particle is a pivotal component for success, and will largely depend both on the coating of the MNPs and the available functional groups on the target moiety [15,16,17,18,19,20,21,22,23,24,25,26]. To improve the sensitivity of sensing devices, signal amplification has been attempted using MNPs. The role of MNPs in our experiment is to pre-concentrate the analyte and, following magnetic separation, remove the undesired effect of the matrix, so that a clean measurable signal can be obtained. In the last decade, MNPs-based EC biosensors [27,28,29,30,31,32,33,34,35,36,37], for salivary diagnostics, have received increasing attention because many substances enter saliva from the blood through transcellular or paracellular diffusion [38]. Consequently, most substances found in blood are also present in saliva. Therefore, saliva is functionally equivalent to serum in reflecting the physiological state of the body but has several advantages over blood [39], as saliva collection is easy, stress free, requires painless sampling, has a simple matrix (less complex than blood), is non-invasive, and offers the possibility of performing real-time monitoring. Saliva has recently been the prominent body fluid for the study of SARS-CoV-2 during the COVID-19 pandemic. Saliva is not only a reliable tool to detect the virus, but can also help in studying the evolution of the virus [40,41,42]. Hence, there are compelling reasons for exploring saliva as a diagnostic and prognostic fluid in heart failure research [43,44] as proposed here. However, saliva is not as constant as blood, as we must keep in mind the change in salivating nature of humans on the sight and smell of food and also depending on the hunger quotient of a particular individual at the specific time. In any case, saliva is there as a potential diagnostic tool due to its ease and non-invasive accessibility along with its abundance of biomarkers, such as genetic material and proteins [45]. 

To learn if salivary diagnosis is applicable in the conditions defined earlier on pre-concentration in saliva we tested tumor necrosis factor alpha (TNF alpha), responsible for a diverse range of signaling events within cells, and Interleukin 10 (IL-10) that plays a central role in limiting host immune response to pathogens, thereby preventing damage to the host. To study the feasibility of saliva and, in these preliminary tests, avoid the variability in concentration of the saliva, we considered adequate to test the immunoreagents in artificial saliva and ensure that there is no cross-reactivity between the immunoreagents used. In this research, we have immobilized anti-TNF-α and anti-IL-10 antibodies, which are present in saliva on MNPs as a proof of concept. The 3D nano-collectors generated were evaluated using UV-vis spectrophotometry (JENWAY 7205) for their ability to pre-concentrate the antigens TNF-α and IL-10, respectively, by employing an external magnetic field. Tests were performed both in phosphate buffered saline (PBS) and artificial saliva (AS) as the matrix. The obtained results have proven that our MNPs can represent a promising tool for rapid pre-concentration of heart failure biomarkers (TNF-α/IL-10) in saliva. Further investigation will be carried out to integrate our MNPs with sensors to increase the sensitivity, selectivity, and decrease matrix effect.

On the other hand, the colloidal stability is a big issue with the MNPs or magnetic nanocomposites, since their advent there remains a big issue surrounding the stability of the MNPs in colloids. The more stable they are in a colloidal state, the less easy it is to use them for magnetic separation, while on the other hand, if they are less stable then they are easier to isolate by external magnetic field application. So, for this a compromise has to be made regarding them and based on their application they are synthesized as needed. In order to improve magnetization, larger particles can be synthesized; these show higher magnetization but much less stability in colloidal state as, due to their large sizes, they have a greater tendency to aggregate. MNPs are more prone to aggregation compared to other nanoparticles and agglomeration is even more significant for MNPs as there exists magnetic dipole–dipole attraction among themselves [46]. In particular, the magnitude of this magnetic dipole–dipole attraction is directly proportional to power 2 of the particle saturation magnetization and power 3 of the particle size. Sedimentation of the agglomerates from its suspension is a challenge [47] that could limit mobility and applicability of MNPs [48] especially in biosensing applications. 

In order to improve stability and also preserve the magnetic properties, the following few things can be done: Sonication, which is a mechanical way and very effective in disaggregating the particles and dispersing them in colloidal state. One of the other methods is using inorganic shell coating like Silica or carbon coating to improve the stability. Also, surface coating with macromolecules like polymers or surfactants can be used for improving stability. Increasing viscosity of the medium can also prevent aggregation as the particles cannot get dispersed easily and travel through the viscous medium, thus preventing aggregation. This can be done by gum gelation or by emulsion formation.

In this paper, one of the first studies done using saliva as a diagnostic fluid for heart failure research is being reported. There lies a novelty in the synthesis procedure in the use of a non-magnetic mechanical stirrer for the coating of SiO_2_ layer on the iron oxide core. This strategy coupled with the concave nature of the reaction flask used results in more homogenized coating of the magnetic core with the Silica layer. The traditional method of using a magnetic bead in this step results in inefficient coating as a major part of the magnetic core becomes attracted to the bead and does not fully participate in the reaction. Using our strategy of a specifically designed mechanical stirrer in the concave flask with Teflon coating on top leads to an increase in the chemical kinetics of the reaction by creating a vortex that leads to homogenized coating with almost negligible reactant losses. Usually, when magnetic nanoparticles tend to aggregate, this causes difficulty in magnetic separation as it is well established that the smaller the size of the particles, the faster is the separation time, while phenomenon like aggregation and flocculation slow down the process [49].

Furthermore, besides a modification in the synthesis procedure of the MNPs and artificial saliva as the fluid for heart failure detection, colloidal stability of MNPs has been studied here with different surfactants and by increasing the viscosity of the medium by using a surfactant. Relevant issues like colloidal stability of MNPs in long-term use for varied applications and how the medium affects their stability are avoided in most studies dealing with MNPs. In this paper, preliminary results of how the stability varies with the change in viscosity of the medium and how difficult it is to extract with an external magnet is reported. Certain common issues with MNPs, which are not reported when its synthesis and application is stated like its long-term usage, stability, and extraction time using external magnets and stirrer systems, are dealt with here.

## 2. Results

**Figure 1:**
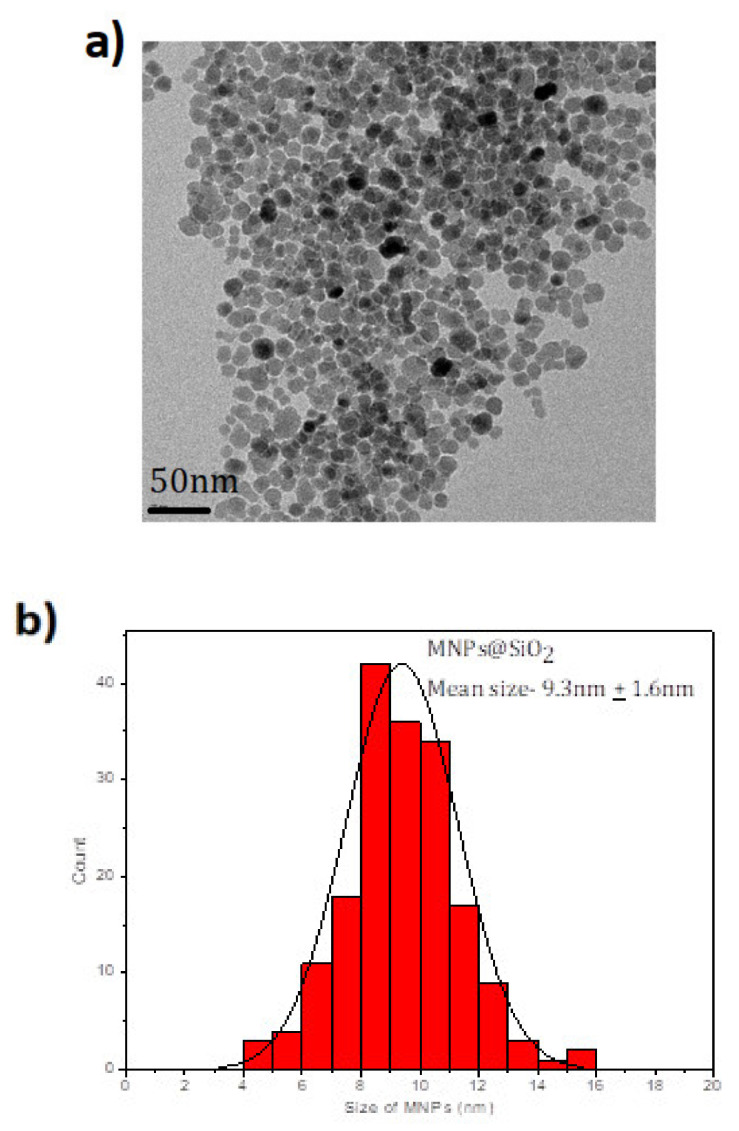
(**a**) The Fe_3_O_4_@SiO_2_ modified magnetic nanoparticles (MNPs) under TEM at 120 kV. They were measured in a copper grid. (**b**) The average size of Fe_3_O_4_@SiO_2_ MNPs is 9.3 ± 1.6 nm.

**Figure 2:**
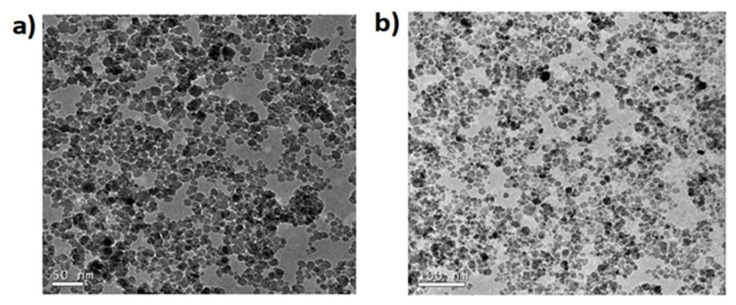
(**a**) Fe_3_O4@SiO_2_-NH_2_ and (**b**) The Fe_3_O_4_@SiO_2_-NH_2_-COOH MNPs under TEM at 120 kV. They were measured in a copper grid. These MNPs were made by using maleic anhydride.

**Figure 3:**
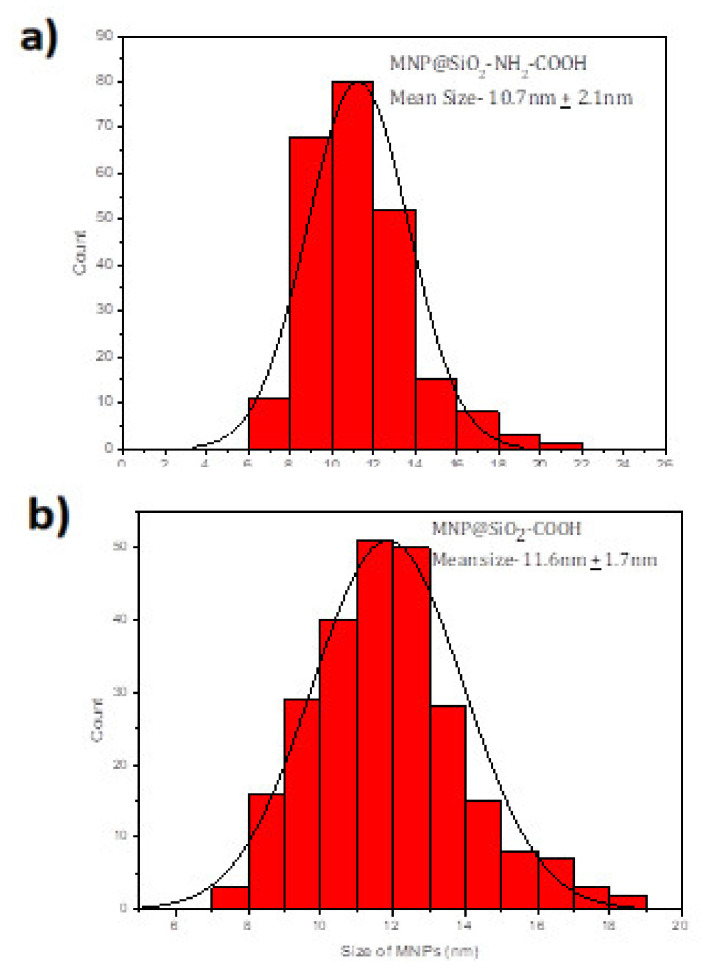
The average size of (**a**) Fe_3_O_4_@SiO_2_-NH_2_ MNPs is 10.7 ± 2.1 nm and (**b**) Fe_3_O_4_@SiO_2_-NH_2_-COOH MNPs is 11.6 ± 1.7 nm.

The syntheses of Fe_3_O_4_@SiO_2_-NH_2_ and Fe_3_O4@SiO_2_-NH_2_-COOH were done using co-precipitation method, followed by Stober process and functionalization [50,51], though the critical point in the synthesis procedure is to use the mechanical stirrer and not the magnetic bead stirring procedure as is commonly used in other colloidal synthesis routes. This is necessary to avoid the MNPs from sticking to the bead, hence the mechanical stirring is necessary to disperse the MNPs in the solution for the reaction to take place. This is used only in the silica coating procedure; after that, magnetic stirring can be used in these MNPs as, after the silica coating, the direct contact to the magnetite core is prevented by the silica shell. After the synthesis of the magnetite core, it is important to first disperse the MNPs using an ultrasonicator before any further reactions. There is a slight increase in the size of the MNPs after silica coating, which is further increased by functionalizing with the NH_2_ and COOH groups. The Fe_3_O_4_@SiO_2_ MNPs are shown in TEM images (see Figure 1a) with the characteristic spherical shape of these MNPs with a mean size of 9.3 ± 1.6 nm (see Figure 1b). When the Fe_3_O_4_@SiO_2_ MNPs are coated with amine and a carboxylic acid, the spherical shape is maintained as Figure 2 shows but the average size increases to 10.7 + 2.1 nm and 11.6 ± 1.7 nm (see Figure 3).

The SEM and EDS techniques provide information about the morphology of the MNPs (Figure S1a) (indicating that the sample is homogenous) and the composition (Figure S2), respectively. The sample showed uniform-sized spheres along with a consistent presence of Fe, Si, and O throughout the sample. The composition was studied using EDS and the analysis was done across the whole sample and it was consistent throughout. Using the EDS technique, it is possible to know the percentage coated by SiO_2_. In this sample, the coating is 1/3 of Si/Fe that is confirmed with the size we get by TEM (Fe_3_O_4_ has a size near 7 nm and Fe_3_O_4_@SiO_2_ has 9.3 nm). Figure S1b shows that the electron diffraction (ED) confirms the cubic spinel structure of the MNPs. This ensures that the magnetization of these MNPs results from the Fe^2+^ ions of the MNPs. Also, further chemical composition was analyzed using the IR spectrum. Fe_3_O_4_@SiO_2_ and Fe_3_O_4_@SiO_2_-COOH was measured with IR and the characteristic peaks of Si-O and C=O was observed in the respective samples (Figure S3) at 1058 cm^−1^ and 1739 cm^−1^, respectively. Further, these MNPs were also characterized using IR before and after being dispersed in PBS, and the peaks of Si-O and C=O were still visible, thus confirming the presence of the functionalized coating of the MNPs (Figure S4).

**Figure 4:**
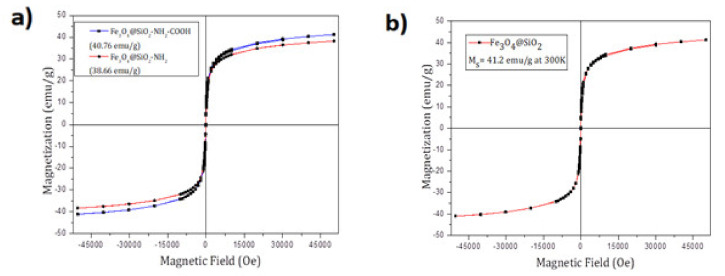
The hysteresis curve of Fe_3_O_4_@SiO_2_-NH_2_ MNPs: 38.66 emu/g, Fe_3_O_4_@SiO_2_-NH_2_-COOH: 40.76 emu/g (**a**) and Fe_3_O_4_@SiO_2:_ 41.2 emu/g (**b**).

Magnetic characterization of the MNPs was carried out in a superconductive quantum interference device (SQUID) magnetometer (Quantum Design MPMS5XL). Magnetization vs. magnetic field measurements were performed at 300K in a 6T field. The samples were prepared using a polycarbonate capsule each filled with 1 mg of Fe_3_O_4_@SiO_2_-NH_2_ or Fe_3_O_4_@SiO_2_-NH_2_-COOH and compacted cotton. We already know that the MNPs show the cubic spinel structure by ED. The magnetic property of iron oxide (Fe_3_O_4_) nanoparticles is dependent on the distribution of Fe ions in octahedral and tetrahedral sites of the spinel structure [52]. The magnetic spins of the ions in the octahedral sites are ferromagnetically coupled to each other and antiferromagnetically coupled with tetrahedral sites. Since the number of Fe^3+^ ions in the octahedral sites and the tetrahedral sites are the same, their magnetic spins cancel out each other. Consequently, the magnetic spins of only Fe^2+^ ions in the octahedral sites contribute to the net magnetic moment in a spinel structure. Figure 4 shows a typical magnetization curve at 300K for superparamagnetic nanoparticles in which neither remnant magnetization (magnetization at zero field, M_R_) nor coercivity (hysteresis loop, H_c_) was observed. The saturation magnetization value of MNPs at 300K was 38.66 emu/g for Fe_3_O_4_@SiO_2_-NH_2_ and 40.76 emu/g for Fe_3_O_4_@SiO_2_-NH_2-_COOH. Also, the magnetization curve for Fe_3_O_4_@SiO_2_ is shown where the saturated magnetization is 41.2 emu/g. (Figure 4) 

The colloidal stability as mentioned at the start is an issue with the MNPs. The MNPs we synthesized were extremely small in size and hence had a tendency to aggregate due to the magnetic dipole–dipole interaction and precipitate over the course of time. This also aided in easily extracting them with the help of an external magnet. To be able to use these MNPs in biosensors, it was important to test their stability and, if possible, increase it without affecting their facile extraction using an external magnet. Five different surfactants were used for this purpose and they are as follows: Citric Acid, Tricaprylylmethyl ammonium chloride, Cetyl trimethyl ammonium chloride (CTAC), Tetrabutyl ammonium chloride, and Dimethyl di-octadecyl ammonium chloride. Out of these five, the last one was successful in providing long-term stability. This was so because it increased the viscosity of the medium and, hence, stopped the MNPs from aggregating. The surfactant prevented the diffusion of the MNPs through the solvent by increasing the viscosity and hence hindering their aggregation. However, this came with a major drawback, as these MNPs were harder to extract using an external magnet. Different concentrations of the surfactant were studied but the trend observed was the same; the more stable they became, the harder it was to extract them. The zeta-potential values obtained for each surfactant is given in Table 1 as well as the different zeta-potential values obtained for different concentrations of dimethyl di-n-octadecyl ammonium chloride in Table 2.

Up until 15 mg of the surfactant, the dispersion was stable, and with time, a small number of MNPs could be extracted by the magnet. However, to obtain them completely dry and free of the surfactant is difficult as it makes the suspension extremely viscous. Figure S10 shows the different time periods needed to extract the MNPs with increasing stability and viscosity of the medium.

The edges of toroidal external magnets have been used in all the cases to extract the magnets as they comprise of the maximum magnetic fields passing through them. The magnets were used on the sidewalls of the vials or reaction flasks to extract the MNPs. Figure S11 shows a photo of the external magnet and the vials used for extraction of the MNPs.

**Figure 5:**
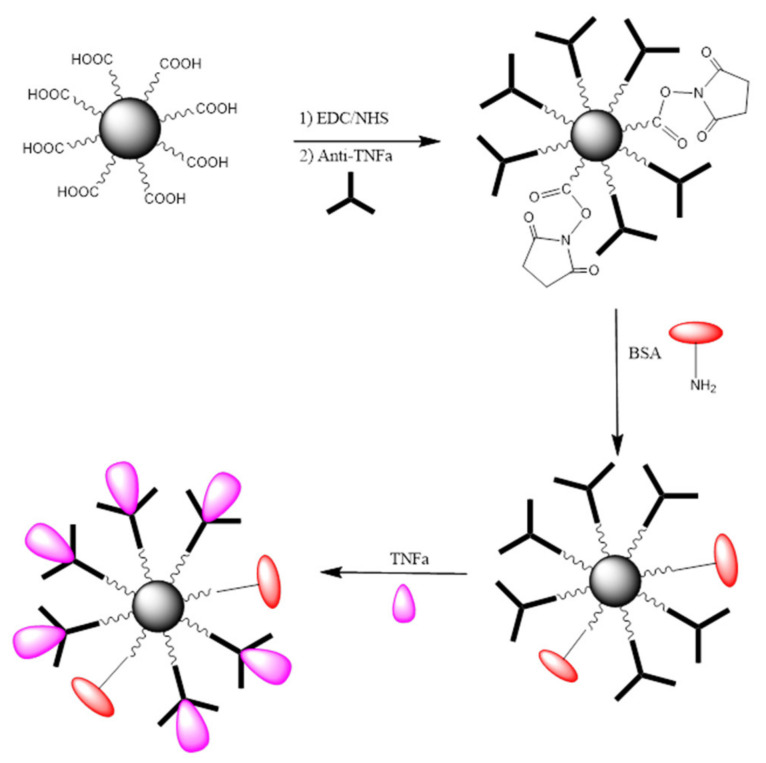
MNPs@SiO_2_-NH_2_-COOH bio-functionalization with anti-TNF-α-antibody. Red: BSA Purple: TNF-α.

To learn about the bio-functionalization efficacy of MNPs with anti-TNF-α antibody, the following experiments were performed: A known amount of MNP@SiO_2_-NH_2_-COOH activated with EDC/NHS was incubated for a fixed period of time with a known concentration of anti-TNF-α antibody. Following a magnetic separation, the supernatant liquid was analyzed by UV-vis spectrophotometry. The bio-functionalization efficacy was evaluated by comparing the absorbance reading to that obtained for the supernatants of the same concentration of anti-TNF-α antibody after incubation with non-activated MNPs. Consequently, the physisorption of antibodies onto MNPs was taken into account and differences in absorbance can only be attributed to the concentration of unreacted antibody remaining in the supernatants. An illustration of the steps involved in the bio-functionalization of MNPs is presented in Figure 5. Firstly, the –COOH groups present on the MNPs surface were activated using a mixture of EDC/NHS followed by the incubation of the activated MNPs with a fixed concentration of antibody to create the complex Antibody-MNPs. Afterwards, the unreacted sites R–COO-NHS were deactivated (BSA was used in the illustration). Finally, the 3D nano-collector MNP@SiO_2_-NH_2_-CO-anti-TNF-α was incubated with the antigen at different concentrations.

**Figure 6:**
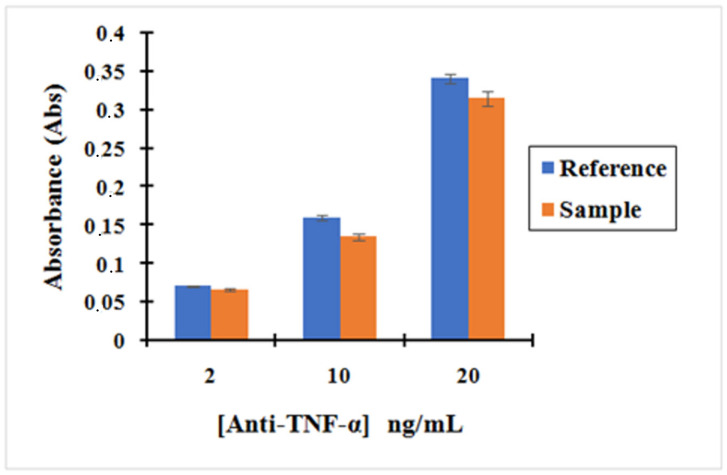
Bar graph showing (blue) the measurement of the absorbance solutions obtained after the immobilization of anti-TNF-α with activated MNPs (with 1-ethyl-3-(dimethylaminopropyl)carbod (EDC)/N-hydroxysuccinimide (NHS)) at different concentrations: 2, 10, and 20 ng/mL; (Orange) the measurement of the absorbance solutions obtained after the immobilization of anti-TNF-α with non-activated MNPs (without EDC/NHS) at different concentration: 2, 10 and 20 ng/mL. Error bars correspond to three replicates per sample.

Figure 6 shows that the absorbance of anti-TNF-α antibody solutions after incubating it with activated MNPs is lower than the absorbance of the anti-TNF-α antibody solutions after incubating with non-activated MNPs. The small difference in the absorbance seen between the Activated MNPs and non-activated MNPs confirms that the activated MNPs binds anti-TNF-α antibody on their surface by the reaction between the amine group of antibody TNF-α and R–COO-NHS groups obtained on the MNPs using EDC/NHS.

This was confirmed by the reproducibility of our three replication measurements. In addition, our bio-functionalized MNPs are vortexed before the removal of supernatant so that the weakly bonded by physical interaction are removed and only the strongly bonded by covalent interaction remain, enabling us to evaluate the effectiveness of our chemistry. These results confirm that the bio-functionalization of MNPs with the antibodies was successfully achieved. Moreover, the concentration of anti-TNF-α in the supernatant decreased by 7%, 16%, and 8% for an initial concentration of 2, 10, and 20 ng/mL, respectively, using the "MNP@SiO_2_-NH_2_-COOH”. Therefore, based on these results, the concentration of anti-TNF-α antibody to run the incubation was fixed at 10 ng/mL to ensure the successful bio-functionalization of MNPs.

The bio-functionalization efficacy was calculated using Beer–Lambert law. Beer–Lambert Law is difficult for quantitative measurement for wavelengths near 200 nm though it has been used here and has provided satisfactory results.

A = εcl(1)

A is the absorbance, ε is the molar attenuation coefficient, l is the optical path length, and c is the concentration of the attenuation species (Equation (1)).
**Concentrations (ng/mL)****2****10****20****Absorbance Sample**0.0660.1350.315**Absorbance Reference**0.0710.1600.341

The methodology that has been used is the following:

A_sample_ = ε C_sample_ l (2)

A_reference_ = ε C_reference_ l (3)

Equation (2)/Equation (3): A_sample_/A_reference_ = C_sample_/C_reference_

C_sample_ = A_sample_ × C_reference_/A_reference_

2 ng/mL

C_sample_ = A_sample_ × C_reference_/A_reference_ = 0.066 × 2/0.071 = 1.85 ng/mL

This is means that the concentration of Antibodies fixed on MNPs is 0.15 ng/mL and the percentage of antibodies fixed in the MNPs is 7%.

10 ng/mL

C_sample_ = A_sample_ × C_reference_/A_reference_ = 0.135 × 10/0.160 = 8.43 ng/mL

This is means that the concentration of Antibodies fixed on MNPs is 1.57 ng/mL and the percentage of antibodies fixed in the MNPs is 16%.

20 ng/mL

C_sample_ = A_sample_ × C_reference_/A_reference_ = 0.315 × 20/0.341 = 18.47 ng/mL

This means that the concentration of Antibodies fixed on MNPs is 1.53 ng/mL and the percentage of antibodies fixed in the MNPs is 8%.

The complex MNP@SiO_2_-NH_2_-CO-anti-TNF-α described in the previous section was used to pre-concentrate TNF-α in PBS. This section details the experiments performed to incubate TNF-α at different concentrations with the complex MNP@SiO_2_-NH_2_-CO-anti-TNF-α and afterwards to measure the supernatants containing the unreacted TNF-α by UV-vis spectrophotometry. Thus, a comparison with the initial concentration of the antigen could be carried out and the percentage of TNF-α, which has been bonded to the complex MNP@SiO_2_-NH_2_-CO-anti-TNF-α, could be assessed.

**Figure 7:**
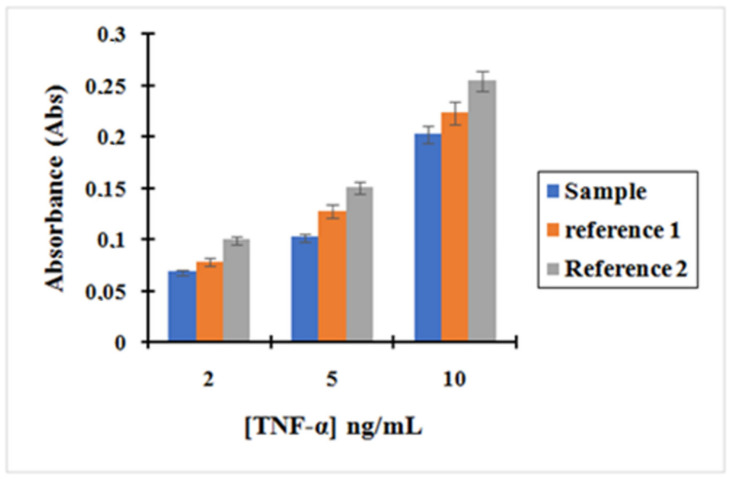
Bar graph showing (blue) the measurement of the absorbance after TNF-α incubation with the complex MNP@SiO_2_-NH_2_-CO-anti-TNF-α at three different concentrations of TNF-α: 2, 5, and 10 ng/L; (Orange) reference 1 represent the measurement of the absorbance of TNF-α after incubation with non-activated MNPs; (grey) Reference 2 represent the measurement of the absorbance of TNF-α after incubation with the initial MNPs.

Figure 7 shows the calibration curve obtained for TNF-α after incubation with the complex MNP@SiO_2_-NH_2_-CO-anti-TNF-α at three different concentrations of TNF-α: 2, 5 and 10 ng/L. The curves, corresponding to reference (reference 1 and 2) to which the former plot was compared, are also shown. Reference (1) depicts supernatants containing unreacted TNF-α after incubation with MNPs that were non-activated with EDC/NHS during the functionalization with anti-TNF-α. Since -COOH groups present on the MNPs were not activated, we did not expect any covalent bonding between anti-TNF-α-MNPs. Therefore, we only expected interaction of TNF-α with those antibodies physisorbed on the MNPs. Reference (2) depicts Supernatants containing unreacted TNF-α after incubation with the initial MNPs that have only been washed four times with 1mL of 10 mM PBS. In this case, the only physisorption of TNF-α on the MNPs was expected.

Based on the results, it can be asserted that the concentration of unreacted TNF-α in the supernatants after incubation with the complex MNP@SiO_2_-NH_2_-CO-anti-TNF-α is lower than the concentration of TNF-α after incubation with the other two references.

As shown in Figure 7, the absorbance decreased following the trend Reference 2 > Reference 1 > Sample. This is consistent with the results expected in Reference 2. The decrease in the amount of TNF-α in the supernatants when compared with its initial concentration can only be attributed to physisorption on the MNPs surfaces. However, in Reference 1, TNF-α can interact with anti-TNF-α antibodies that have been adsorbed to the surface of the MNPs. This effect is shown by a decrease in the absorbance read when compared to Reference 2. Finally, in “Sample”, the complex MNP@SiO_2_-NH_2_-CO-anti-TNF-α binds the antigen TNF-α more effectively as antibodies are both covalently bonded and physisorbed to the MNPs. Therefore, the pre-concentration effect when incubating TNF-α with “Sample” is much more important. 

Thus, we can confirm these two hypotheses:

(1) There is an effective coupling between MNPs and anti-TNF-α antibody when the MNPs were activated using EDC/NHS.
(2) The complex MNP@SiO_2_-NH_2_-CO-Anti-TNF-α can bind the antigen TNF-α and it can be followed by UV-vis spectrophotometry.

The previous experiment as described above was tested using artificial saliva as a matrix. First, a stock solution of artificial saliva (AS) was prepared by dissolving 0.6 g/L Na_2_HPO_4_, 0.6 g/L anhydrous CaCl_2_, 0.4 g/L KCl, 0.4 g/L NaCl, 4 g/L mucin, and 4 g/L urea (purchased from Sigma-Aldrich, France) in deionized water. The pH was adjusted to 7.2 by adding NaOH and it was stored at 4 °C until use.

A dilution profile was performed by UV-vis spectrophotometry to find out the optimal dilution range of artificial saliva at which the matrix effect is removed. For this purpose, AS was diluted using 10 mM PBS to obtain saliva/PBS ratios of 1/1, 1/10, 1/100, 1/500, and 1/1000. The results are shown in Figure S5. Based on the results obtained, AS diluted with PBS at a ratio 1/500 was chosen as a matrix. Afterwards, a new calibration curve of TNF-α using artificial saliva diluted 1/500 as the matrix was carried out at different concentrations of TNF-α: 2, 5, and 10 ng/L. The results are shown in Figure S6 and confirmed the dependency of the absorbance with the concentration of TNF-α.

In the first stage, the complex MNP@SiO_2_-NH_2_-CO-anti-TNF-α was obtained as described above. Subsequently, TNF-α was prepared in AS diluted in PBS 1/500 at three different concentrations: 2, 5, and 10 ng/mL. The solutions prepared were incubated with the complex MNP@SiO_2_-NH_2_-CO-Anti-TNF-α as described above and the supernatants containing the unreacted TNF-α were measured by UV-vis spectrophotometry.

Once again, the solution used for the reference was based on the supernatants containing unreacted TNF-α after incubation with MNPs that were non-activated with EDC/NHS during the functionalization with anti-TNF-α antibody.

**Figure 8:**
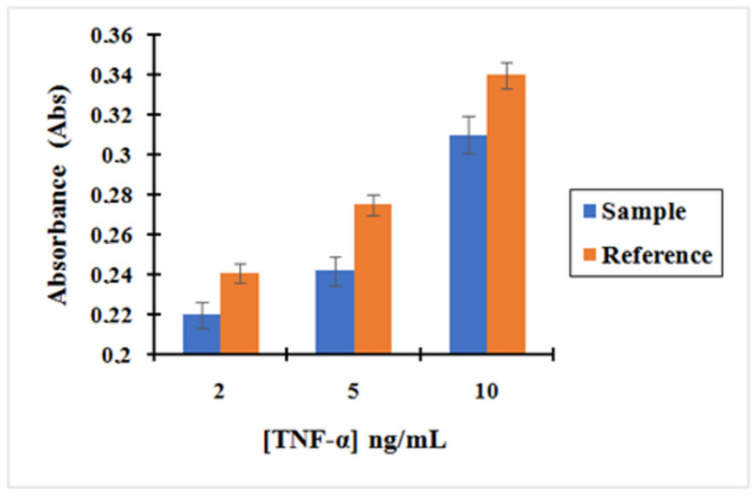
Bar graph showing (blue) the measurement of the absorbance after TNF-α incubation with the complex MNP@SiO_2_-NH_2_-CO-anti-TNF-α at three different concentrations of TNF-α: 2, 5, and 10 ng/L; (Orange) the measurement of the absorbance of TNF-α after incubation with no activated MNPs. Background fixed in artificial saliva at 1/500. Error bars correspond to three replicates per sample.

Based on these results shown in Figure 8, we could confirm that the concentration obtained for the supernatant containing the unreacted amount of TNF-α after incubation with the nano-collector MNP@SiO_2_-NH_2_-CO-anti-TNF-α was lower than the supernatant of TNF-α after incubation with non-activated MNP (without activation with EDC/NHS) used as a reference. Therefore, it can be concluded that the pre-concentration effect when incubating TNF-α with the complex MNP@SiO_2_-NH_2_-CO-anti-TNF-α is much more important. The concentration of TNF-α decreased by 9%, 12%, and 9% when compared to the initial concentration of 2, 5, and 10 ng/mL, respectively, using the MNP@SiO_2_-NH_2_-COOH.

The same experiment described with TNF-α was repeated for the cytokine IL-10 and its corresponding anti-IL-10 antibody. First, the concentration range at which IL-10 can be measurable by UV-vis spectrophotometry in a fixed background of AS diluted 1/500 with PBS was obtained. In the UV region, IL-10 was shown to absorb 10 times less than TNF-α for the same concentration. Consequently, the results showed a working range of IL-10 at a concentration between 10 and 100 ng/mL (Figure S7).

The concentration of IL-10 decreased by 7%, 6%, and 4% when compared to the initial concentration of 10, 40, and 100 ng/mL, respectively, using MNP@SiO_2_-NH_2_-COOH. Once again, the results demonstrated the ability of the complex antibody-MNPs to pre-concentrate the antigen.

A comparative study of different deactivating compounds was carried out. The aim was to assess the adsorption effect of the TNF-α antigen when using different chemicals/proteins for the deactivation step performed after bio-functionalization of MNPs with an antibody. For this purpose, four different deactivation mixtures were used: 0.1% bovine serum albumin (BSA) in 10 mM PBS, ethanolamine 0.1% in 10mM PBS, poly (ethylene glycol) methyl ether amine (PEG-NH_2_) 0.1% in 10 mM PBS, and ethanolamine cyanoborohydride (NaBH_3_CN) 0.1% in 10 mM PBS.

The comparative study was performed using activated MNPs with EDC/NHS: MNPs@SiO_2_-NH_2_-COOH. They were reacted with anti-TNF-α antibody at a concentration of 10 ng/mL, which was chosen as the antibody for the bio-functionalization of MNPs. The experimental procedure concerning the MNPs bio-functionalization was described above.

**Figure 9:**
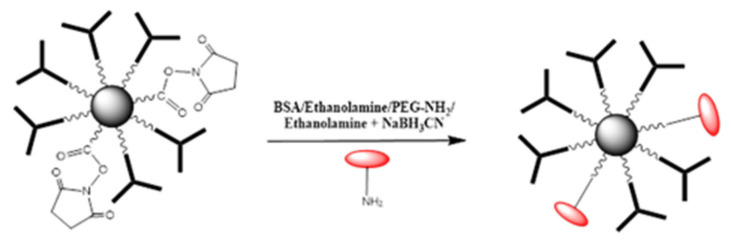
Deactivation of remaining R-CO-NHS groups using BSA/Ethanolamine/PEG-NH_2_/Ethanolamine + NaBH_3_CN.

The deactivation protocol was the same as described above. The MNPs were activated using a mixture of EDC/NHS. Then, anti-TNF-α antibody was added to the activate MNPs. Afterwards, the deactivating solution was changed consecutively as aforementioned. As an illustration, the deactivation of remaining R-CO-NHS groups using BSA/Ethanolamine/PEG-NH_2_/Ethanolamine + NaBH_3_CN is presented in Figure 9.

**Figure 10:**
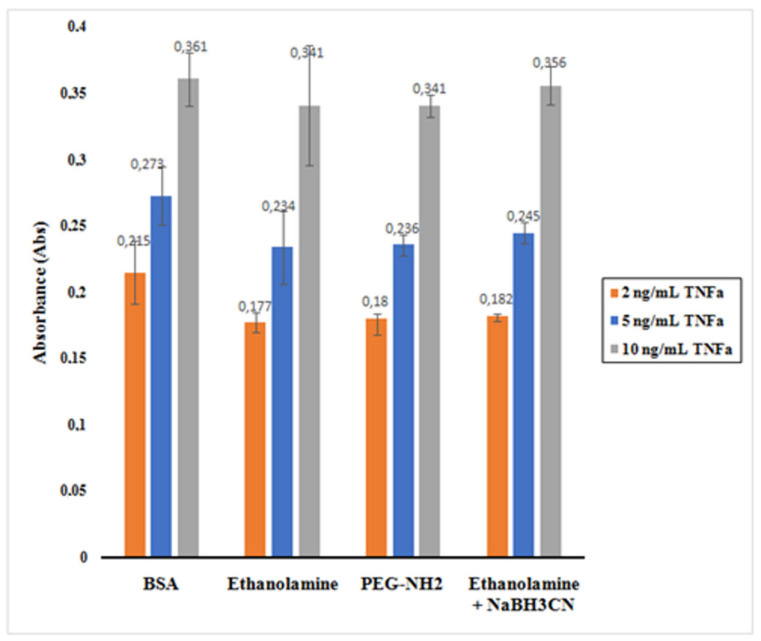
Impact of different deactivation mixtures (BSA, ethanolamine, PEG-NH_2_, ethanolamine + NaBH_3_CN) to the physisorption effect of TNF-α on 3D nano-collector MNP@SiO_2_-NH_2_-CO-anti-TNF-α. Error bars correspond to three replicates per sample.

The histogram presented in Figure 10 shows the absorbance reading for the supernatants containing the unreacted TNF-α after incubation with the nano-collector MNP@SiO_2_-NH_2_-CO-anti-TNF-α for the different deactivating mixtures.

It is observed that BSA appeared to be the best among the mixtures assessed. When 0.1% of BSA in PBS was used as deactivating mixture, TNF-α was less physisorbed to the nano-collector leading to a higher absorbance as the total amount of unreacted TNF-α remained relatively high. The ethanolamine + NaBH_3_CN mixture also indicated similar behavior. However, in the case of ethanolamine and PEG-NH_2_, the absorbance reading was lower as a result of a decreased concentration of unreacted TNF-α.

**Figure 11:**
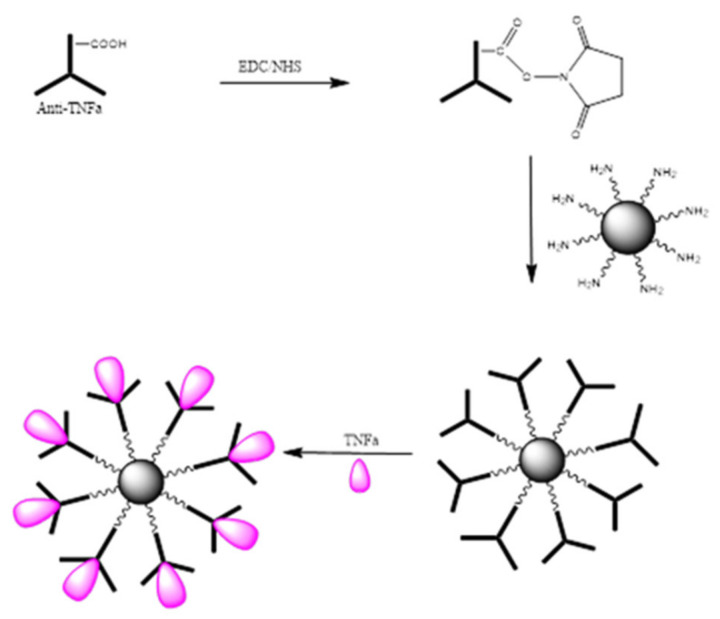
MNP@SiO_2_-NH_2_ bio-functionalization with anti-TNF-α antibody.

The bio-functionalization of MNPs with anti-TNF-α antibody at different concentrations was carried out. An illustration of the steps involved in the bio-functionalization of MNPs is presented in Figure 11. Firstly, the –COOH groups present on the anti-TNF-α antibody surface were activated using a mixture of EDC/NHS followed by the incubation of MNPs to the activated anti-TNF-α antibody to create the complex antibody-MNPs. Finally, the 3D nano-collector MNP@SiO_2_-NH-anti-TNF-α was incubated with TNF-α.

**Figure 12:**
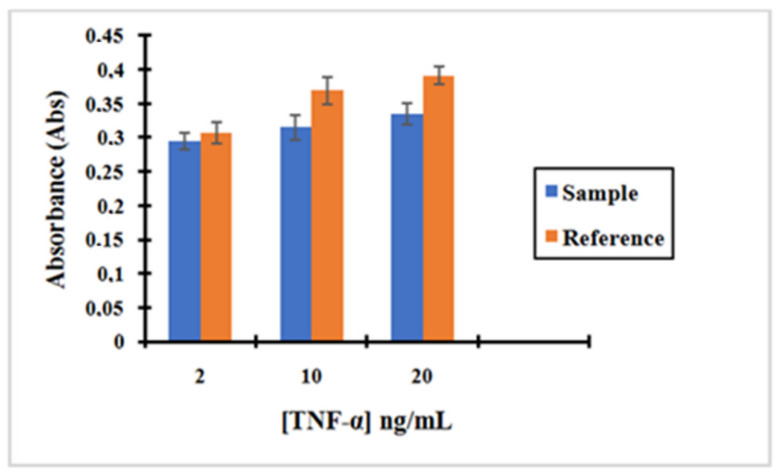
Bar graph showing (blue) the measurement of the absorbance after TNF-α incubation with the complex MNP@SiO_2_-NH_2_-CO-anti-TNF-αat three different concentrations of TNF-α: 2, 5, and 10 ng/L; (Orange) the measurement of the absorbance of TNF-α after incubation with no activated MNPs. Background fixed in artificial saliva at 1/500. Error bars correspond to three replicates per sample.

Based on the results shown in Figure 12, we can confirm again that the concentration obtained for the supernatant containing the unreacted amount of TNF-α after incubation with the nano-collector MNP@SiO_2_-NH-anti-TNF-α is lower than the supernatant of TNF-α after incubation with non-activated anti-TNF-α antibody (without activation with EDC/NHS) used as a reference. Therefore, it can be concluded that the pre-concentration effect when incubating TNF-α with the complex anti-TNF-α-MNPs is much more important. The concentration of TNF-α decreased by 5%, 16%, and 17% when compared to the initial concentration of 2, 5, and 10 ng/mL, respectively.

## 3. Materials and Methods

### 3.1. Materials

#### 3.1.1. Instrumentation

A magnetic rack purchased from Sigma Aldrich (Lyon, Auvergne-Rhône-Alpes, France) and a tube revolver/rotator purchased from Thermo Fisher Scientific (Waltham, Massachusetts, U.S) were used during the bio-functionalization process of MNPs. All experiments for indirect measurements of the biomarkers were carried out at room temperature (22 ± 2 °C). The indirect measurements were carried out using UV-vis spectrophotometry (JENWAY 7205) purchased from Jeulin (Evreux, Normandy, France). Transmission electron microscopy (TEM) studies were carried out using JEOL JEM 1210 at 120 kV. Scanning electron microscopy (SEM) and energy dispersive X-ray spectroscopy (EDX) analysis was done using the QUANTA FEI 200 FEG-ESEM device. The solid sample was analyzed for this. Magnetization hysteresis cycle was measured using Quantum Design MPMS-XL system at 300K with a maximum of 60 kOe.

#### 3.1.2. Chemicals and Reagents

Sodium chloride (NaCl, purity ≥ 99.5%), potassium chloride (KCl, purity 99.0–100.5%), sodium phosphate dibasic (Na_2_HPO_4_, PharmaGrade), magnesium nitrate (Mg(NO_3_)_2_, purity 98%), calcium chloride (CaCl_2_, purity ≥ 93%), mucin (from pork stomach extract, type II), sodium hydroxide (NaOH, pellets, purity ≥ 98%), Urea, phosphate buffer solution (PBS) tablets, hydrochloric acid (HCl, ACS reagent, 37%), 1-ethyl-3-(dimethylaminopropyl) carbodiimide (EDC), N-hydroxysuccinimide (NHS), bovine serum albumin (BSA), ethanolamine, poly (ethylene glycol) methyl ether amine (PEG-NH_2_), and sodium cyanoborohydride (NaBH_3_CN) were purchased from Sigma-Aldrich (France). Anti-TNF-α antibody (Catalog number: MAB610-500), Anti-IL-10 antibody (Catalog number: MAB217-500), TNF-α (Catalog number: 210-TA), and IL-10 (Catalog number: 217-IL-050) were purchased from R&D Systems (France). Millipore Milli-Q nanopure water (resistivity > 18 MΩ cm) was produced bya Millipore Reagent Water System (France). The PBS buffer used in this study was prepared by dissolving PBS tablets in the nanopure water as indicated by the supplier by yielding 0.01 M phosphate buffer (pH 7.4) containing 0.0027 M potassium chloride and 0.137 M sodium chloride. All reagents used in the present work for the synthesis of magnetic nanoparticles and its functionalization were obtained from Aldrich Chemical Co and were used without further purification. Aqueous ammonia (30% as NH_3_) was purchased from Panreac AppliChem and used as received.

### 3.2. Procedure of Synthesis of MNPs

The synthetic procedure has been modified and adapted to our needs. The procedure used for synthesis has been a modification of the procedure used by Hassani et al. for Stöber process and Krajl et al. for the amine and the consecutive COOH functionalization [50,51].

#### 3.2.1. Synthesis of Fe_3_O_4_ Nanoparticles

First, 25 mL of a 15M solution of sodium hydroxide (NaOH) in distilled water was made. Then, a mixture of Iron (II) chloride tetrahydrate (FeCl_2_.4H_2_O) (2 g) and Iron (III) chloride hexahydrate (FeCl_3_.6H_2_O) (5.2 g) was added to 25 mL of distilled water. The NaOH solution previously made was added slowly to the mixture of the iron salts with vigorous stirring. Then, 1 mL of concentrated hydrochloric acid (HCl) was added dropwise along with vigorous stirring to make a black solid product. The resultant mixture was heated using an oil bath for 6 h at 80 °C. The black magnetite solid MNPs were isolated using an external magnet and washed three times with distilled water and then dried at 80 °C for overnight. The Iron oxide (Fe_3_O_4_) nanoparticles were formed. In the step for the addition of the NaOH, the base is added slowly to the iron salts to obtain smaller sized MNPs while if the iron salts are added directly into the base the sizes of the particle obtained are larger, so it was avoided.

#### 3.2.2. Preparation of the Fe_3_O_4_@SiO_2_ Core–Shells

First, 5 mmol of Fe_3_O_4_ as-synthesized was dispersed in a mixture of ethanol (100 mL) and distilled water (25 mL) for 30 min. Then, 3 mL of NH_3_ was added followed by the dropwise addition of tetraethoxysilane (TEOS) (1.7 mL). This solution was stirred mechanically for 8 h at room temperature (r.t). Then, the product Fe_3_O_4_@SiO_2_ was separated using an external magnet, was washed three times with distilled water and three times with ethanol, and then dried at 80 °C overnight. 

#### 3.2.3. Preparation of Carboxylic Acid and Amine Functionality on Fe_3_O_4_@SiO_2_ Nanoparticles [Fe_3_O_4_@SiO_2_@NH_2_ and Fe_3_O_4_@SiO_2_@NH_2_-COOH]

First, 125 mg of Fe_3_O_4_@SiO_2_ was suspended using an ultra-sound bath in 45 mL of ethanol. Then, 5 mL of 3-(2-aminoethylamino) propyl methyl dimethoxy silane (APMS) was dissolved in 20 mL ethanol (20 wt%), and was added to the suspension of Fe_3_O_4_@SiO_2_ in ethanol and stirred vigorously. The pH of the solution was then maintained at 11 using tetramethyl ammonium hydroxide (TMAH). After that, the solution was stirred for 5 h at 50 °C. Then, the Fe_3_O_4_@SiO_2_-NH_2_ was obtained by magnetic separation, and was washed with distilled water thrice. Some of the particles were not isolated at this stage, and were kept in dispersed form, so sodium chloride (NaCl) (20 mg in 50 mL of deionized water) was added to the dispersion and with this, extra precipitation was achieved. After redispersion, these particles were magnetically decanted and washed thrice. Both crops were mixed together and dried at the oven at 80 °C. Then, 100 mg of Fe_3_O_4_@SiO_2_-NH_2_ was dispersed in 45 mL dry dimethylformamide (DMF) and 60 mg of succinic anhydride was added to this suspension. The mixture was then stirred with a magnetic stirrer for 22 h at room temperature r.t. The MNPs were then washed thrice with acetone, separated with a magnet, and dried under vacuum. The Fe_3_O_4_@SiO_2_-NH_2_-COOH MNPs were ready to be used.

#### 3.2.4. Antibody (TNF-α) Grafted at the Surface of MNP@SiO_2_-NH_2_-COOH

(I)- 100 µL, 0.5% solid content of the mother solution containing MNPs@SiO_2_-NH_2_-COOH in water were washed following magnetic separation with 1 mL of 10 mM PBS. The washing was repeated three times to ensure all the residues were washed out. The activation of carboxylic acid groups present on the MNPs was achieved by incubation of MNPs in a 500 µL mixture of EDC/NHS (250 µL of 100 mM EDC + 250 µL of 100 mM NHS both prepared in 10 mM PBS) at r.t, and under soft stirring using tube revolver/rotator for 90 min. Then, the remaining EDC/NHS was removed and the MNPs were washed for three consecutive times using 1 mL of cold HCl 1 mM. Subsequently, 500 µL of purified anti-TNF-α antibody was added. Accordingly, three different concentrations of antibody (2, 10, and 20 ng/mL) were used to assess the impact of the concentration on the bio-functionalization efficacy. The activated mixture of MNPs + anti-TNF-α antibody was slowly stirred at r.t for 2 h 30 min. Finally, the complex MNPs@SiO_2_-NH_2_-CO-Antibody was obtained. It was further immobilized using the magnetic rack and the supernatants were collected and measured by UV-vis spectrophotometry.

(II)- For reference, the same procedure was repeated. However, the activation step with EDC/NHS was skipped. Consequently, the supernatants collected after incubation of anti-TNF-α antibody with MNPs correspond to the initial concentration of antibody minus the number of antibodies that were physisorbed to the MNPs. This phenomenon also occurs in (I)-. Therefore, the final concentration measured in the supernatants corresponding to (I) represents the initial concentration of anti-TNF-α antibody minus the amount of anti-TNF-α antibody covalently bonded to the MNPs minus the amount of anti-TNF-α antibody physisorbed. In summary, the absorbance read for (I) and (II) are as follows (Equation (4) and Equation (5)):

Af(I) = A0 − A (MNP@SiO_2_-NH_2_-CO-anti-TNF-α) − A (phy) (4)

Af(II) = A0 − A (phy)(5)

where Af(I) and Af(II) correspond to the absorbance read of the supernatants obtained in (I) and (II), respectively. A0 represents the absorbance corresponding to the initial concentration of antibody, while A (phy) indicates the absorbance corresponding to the number of antibodies physisorbed onto the MNPs, and A (MNP@SiO_2_-NH_2_-CO-anti-TNF-α) to the absorbance corresponding to the number of antibodies covalently bonded to the MNPs. Figure S14 shows an illustration of the experimental procedure carried out for the bio-functionalization of MNPs with anti-TNF-α antibody.

#### 3.2.5. Antibody Interleukin-10 (IL-10) Grafted at the Surface of MNP@SiO_2_-NH_2_-COOH

First, 100 µL, 0.5% solid content of the mother solution containing MNPs@SiO_2_-NH_2_-COOH in water were washed following magnetic separation with 1 mL of 10 mM PBS. The washing was done thrice to ensure all the residues were washed out. The activation of carboxylic acid groups present on the MNPs was achieved by incubation of MNPs in a 500 µL mixture of EDC/NHS (250 µL of 100 mM EDC + 250 µL of 100 mM NHS both prepared in 10 mM PBS) at r.t, and under soft stirring using tube revolver/rotator for 90 min. Then, the remaining EDC/NHS was removed and the MNPs were washed for three consecutive times using 1 mL of cold HCl 1 mM. Subsequently, 500 µL of purified Anti-IL-10antibody (10 ng/mL) was added. The activated mixture of MNPs + Anti-IL-10 was slowly stirred at r.t for 2 h 30 min. Finally, the complex MNPs@SiO_2_-NH_2_-CO-Anti-IL-10 was obtained.

#### 3.2.6. Indirect Detection of TNF-α in PBS. Pre-Concentration of TNF-α onto the Complex MNP@SiO_2_-NH_2_-CO-anti-TNF-α

(I) The complex MNP@SiO_2_-NH_2_-CO-anti-TNF-α obtained was washed with 1 mL of 10 mM PBS. A magnetic rack was placed to immobilize the complex and allow the discharge of supernatants and the added PBS. The cleaning was repeated three times to ensure all the residues were washed out. Afterwards, the unreacted sites were deactivated using a solution of 0.1% BSA in PBS (500 µL in each Eppendorf). The mixture was stirred (33 rpm) using tube revolver/rotator at r.t for 45 min. Then, the unreacted BSA was removed and the complex was washed three times with 1 mL of 10 mM PBS. Again, magnetic separation was done. At this point, the complex was incubated with TNF-α at three different concentrations: 2, 5, and 10 ng/mL. Incubation was performed at r.t and stirred at 33 rpm for 45 min. Finally, after 45 min, the supernatants containing the unreacted TNF-α were collected and the absorbance was measured for the respective concentrations. Figure S15 shows an illustration of the experimental procedure carried out for the Pre-concentration of TNF-α using the complex MNP@SiO_2_-NH_2_-CO-anti-TNF-α.

(II) For the reference, two approaches were compared:

Reference (1) Supernatants containing unreacted TNF-α after incubation with MNPs that were NOT ACTIVATED with EDC/NHS during the functionalization with anti-TNF-α. Since -COOH groups present on the MNPs were not activated, we do not expect any covalent bonding anti-TNF-α-MNPs. Therefore, we only expect interaction of TNF-α with those antibodies physisorbed on the MNPs. 

Reference (2) Supernatants containing unreacted TNF-α after incubation with the initial MNPs that have only been washed four times with 1mL of 10 mM PBS. In this case, the only physisorption of TNF-α on the MNPs is expected.

#### 3.2.7. Indirect Detection of IL-10 in Artificial Saliva. Pre-Concentration of IL-10 onto the Complex MNP@SiO_2_-NH_2_-CO-Anti-IL-10

The complex MNP@SiO_2_-NH_2_-CO-Anti-IL-10 obtained was washed with 1 mL of 10 mM PBS. A magnetic rack was placed to immobilize the complex and allow the discharge of supernatants and the added PBS. The cleaning was done thrice to ensure all the residues were washed out. Afterwards, the unreacted sites were deactivated using a solution of 0.1% BSA in PBS (500 µL in each Eppendorf). The mixture was stirred (33 rpm) using tube revolver/rotator at room temperature for 45 min. Then, the unreacted BSA was removed and the complex was washed three times with 1 mL of 10 mM PBS. Again, magnetic separation was done. At this point, the complex was incubated with IL-10 at three different concentrations: 10, 40, and 100 ng/mL. Incubation was performed at r.t and stirred at 33 rpm for 45 min. Finally, after 45 min, the supernatants containing the unreacted IL-10 were collected and the absorbance was measured for the respective concentrations. 

#### 3.2.8. Bio-Functionalization of MNPs Decorated with Amine Groups “MNP@SiO_2_-NH_2_”

First, 100 µL of the anti-TNF-α antibody at 10 ng/mL were activated by the incubation in a 500 µL mixture of EDC/NHS (250 µL of 100 mM EDC + 250 µL of 100 mM NHS both prepared in 10 mM PBS) at r.t, and under soft stirring for 90 min. Then, the activated anti-TNF-α antibody was added to the MNP@SiO_2_-NH_2_ (100 µL, solid content 0.5%) at r.t, and under soft stirring, for 2 h 30 min to create the complex MNPs@SiO_2_-NH-Antibody. Afterwards, MNPs were washed for three consecutive times using 1 mL of cold HCl 1 mM, followed by washing for three times using 1 mL of PBS 10 Mm. Finally, the complex was used for the pre-concentration with different concentrations of TNF-α (2, 10, 20 ng/mL) and the supernatants were collected and measured by UV-vis spectrophotometry.

### 3.3. Calibration Curve of Anti-TNF-α Antibody and TNF-α in PBS

In the first stage, a calibration curve was obtained in PBS in order to determine the optimal range of concentration at which both TNF-α and Anti-TNF-α can be analyzed by UV-vis spectrophotometry.

#### 3.3.1. Calibration Study of Anti-TNF-α Antibody

Firstly, Anti-TNF-α antibody was prepared in PBS 10 mM at three different concentrations: 20, 100, and 200 ng/mL; 10 mM solution of PBS was used as blank. All the measurements were carried out in duplicates.

As shown in Figure S16, a peak for Anti-TNF-α was found at λ = 206 nm. However, we also observed that the absorbance is saturated at concentrations of antibody higher than 100 ng/mL. Therefore, the experiment was repeated using a dilution factor Anti-TNF-α antibody/PBS 1/10. Figure S17 shows the UV-vis spectrum of Anti-TNF-α at concentrations of 2, 10, and 20 ng/mL.

Here, the absorbance increased linearly with the concentration of antibody in conformity with the Beer–Lambert’s law (A = εcl). The saturation effect disappeared and therefore the concentration range was fixed between 2 and 20 ng/mL. Measurements were repeated three times and the calibration curve obtained is shown in Figure S18.

#### 3.3.2. Calibration Study of TNF-α

Likewise, a calibration study was carried out to assess the concentration range at which the antigen TNF-α is measurable by UV-vis spectrophotometry. For this purpose, TNF-α was prepared in PBS 10 mM at different concentrations: 2, 5, and 10 ng/mL. A 10mM solution of PBS was used as blank. All the measurements were carried out in duplicates. A peak for TNF-α was found at λ = 205 nm. In this case, no saturation was observed and the concentration range of antigen was fixed from 2 to 10 ng/mL. The calibration curve obtained is shown in Figure S19.

## 4. Conclusions

We would like to conclude that MNPs with Fe_3_O_4_ core has been synthesized and coated with a layer of SiO_2_ using the Stöber process. A good SiO_2_ layer homogeneity has been achieved using an overhead mechanical stirrer with a glass bar containing two blades in a beaker with a Teflon lid to prevent alcohol evaporation while other processes have been performed using typical magnetic stirrers with a magnetic bead. The SiO_2_ coating has been decorated with –NH_2_ and –COOH free end groups. The MNPs thus produced were Fe_3_O_4_@SiO_2_, Fe_3_O_4_@SiO_2_-NH_2_, and Fe_3_O_4_@SiO_2_-NH_2_-COOH that show a gradual increase in size with the additional functionalized layers. Colloidal stability of the MNPs have also been studied using different surfactants and observing the change in the zeta-potential values of the colloids. The –COOH and –NH_2_ MNPs have been further functionalized with anti-TNF-α and anti-IL-10 antibodies using (EDC) and (NHS) reagents. The magnetic conjugates produced can be easily extracted using an external magnet. These MNPs have been prepared for testing the tumor necrosis factor alpha (TNF-α), while the others have been prepared for Interleukin-10 (IL-10). To have the highest selectivity towards TNF-α, and IL-10, the non-functionalized MNPs surface sites have been neutralized with the BSA protein. The anti-TNF-α and anti-IL-10 antibodies MNPs have been incubated with TNF-α and IL-10, respectively, in PBS, and after magnetic separation the supernatant solution has been tested for TNF-α and IL-10, respectively, in PBS. They have been measured by UV-vis spectrophotometry where a lower absorbance reading indicates better MNPs capture. The supernatant containing unreacted TNF-α that had been incubated with MNPs whose –COOH reagents has been used as the main reference. It had not been activated with EDC/NHS, although anti-TNF-α or anti-IL-10 antibodies had been added (named Reference 1). Although these did not presumably have any bonded anti-TNF-α antibody or anti-IL-10 antibody, they may indeed have some anti-TNF-α antibody or anti-IL-10 antibody physisorbed, thus interacting with TNF-α or IL-10. Further, there could have been the physisorption on the MNPs by the very same TNF-α and IL-10. To test this, TNF-α and IL-10 were incubated with MNPs before EDC/NHS activation (named Reference 2). Without any exception, the MNPs incorporating the antibodies gave the lowest value of TNF-α and IL-10 in solution, which implies their highest uptake by the MNPs, whereas the highest value of TNF-α and IL-10 was for Reference 2. All this implies that antibodies can be adsorbed, in part, on the surface of silica, a factor that has to be taken in consideration. All these studies were done in PBS, but it was sought to use saliva as the body fluid. To apply the UV-vis spectrophotometry technique it was observed that the saliva had to be diluted with PBS to record the amount of TNF-α and IL-10. Dilution was necessary to 1:500 with PBS. By using Fe_3_O_4_@SiO_2_-NH_2_-CO-anti-TNF-α antibody or anti-IL-10 antibody the pre-concentration process for TNF-α and IL-10 was clear.

When the coupling with the anti-TNF-α antibody or anti-IL-10 antibody occurs, not all activated COOH generate the desired CO-anti-TNF-α or anti-IL-10; some remain “unreacted” and to avoid later reaction with TNF-α or IL-10 that would obscure the reaction, it is necessary to use one deactivating agent. Additionally, these deactivating agents also fill the cavities in the MNPs preventing their occupation by physisorbed antibodies or antigens. Among the several compounds/mixtures utilized like BSA, ethanolamine, ethanolamine+ sodium cyanoborohydride (NaBH3CN), and poly(ethylene glycol) methyl ether amine (PEG-NH2), the BSA and ethanolamine + NaBH3CN were found to be the most efficient. To conclude, in this work, we demonstrate the successful synthesis of two different kind of MNPs (Fe3O4@SiO2-NH2-COOH; Fe3O4@SiO2-NH2) as well as the characterization of these MNPs using different techniques. Then, the high selectivity of these MNPs to pre-concentrate two kind of Heart failure biomarkers: TNF-α (2–20 ng/mL) and IL-10 (10–100 ng/mL) in a complex medium (artificial saliva) after five hours of biofunctionalization process was studied. 

In this paper, other than the use of the new set-up using mechanical stirrer with glass blades having a Teflon cover for homogeneous coating of the Silica layer, it reports one of the first studies done using saliva as a diagnostic fluid for heart failure research. Furthermore, colloidal stability of MNPs has been studied here with different surfactants and by increasing the viscosity of the medium by using a surfactant. Preliminary results of how the stability varies with the change in viscosity of the medium and how difficult it is to extract with an external magnet is reported here. This work tries to point out the little concern that exists on the numerous studies of MNPs about taking into consideration the time required for magnetic extraction, or the medium and how it affects the magnetic extraction and the stability of the MNPs for long time shell storage in case they are intended to be used in automatic machines without trained personnel. Further, it deals with the kind of stirrers needed for synthesizing MNPs and that simple UV-vis spectroscopy could be used to determine the loading of MNPs. This paper aims towards showing certain basic fundamental things needed for using MNPs, rather than just reporting a synthesis of MNPs and the detection of antigens. Some basic fundamentals of magnetic nanoparticles, which are mostly excluded from the papers dealing with such topics have been addressed here.

## Figures and Tables

**Table 1:** Stability of Fe_3_O_4_@SiO_2_-NH_2_ with different surfactants.

Surfactant	Zeta-Potential (mV)
Citric acid	15.6
Tricaprylyl methyl ammonium chloride	26.8
CTAC	22.6
Tetrabutyl ammonium chloride	36.9
Dimethyl di-n-octadecyl ammonium chloride	54.2

**Table 2:** Stability of Fe_3_O_4_@SiO_2_-NH_2_ with different amounts of dimethyl di-n-octadecyl ammonium chloride.

MNPs (wt) (mg/mL)	Dimethyl di-n-octadecyl Ammonium Chloride (wt) (mg)	Zeta-Potential (mV)
**0.5**	2.5	15.4
**0.5**	5.0	18.9
**0.5**	10	28.4
**0.5**	15	30.2
**0.5**	20	38.7
**0.5**	25	44.6
**0.5**	30	50.2

## Supplementary Materials

The following are available online at https://www.mdpi.com/1420-3049/25/17/3968/s1, It includes the Results and Discussion Images (Figure S1 to S19, SEM Image, ED pattern, EDX spectrum, IR studies, UV-vis spectra, calibration curves, reaction mechanism, time studies for magnetic extraction and image of MNPs).

## References

[1] Pankhurst A., Connolly J., Jones S.K., Dobson J. (2003). Applications of magnetic nanoparticles in biomedicine. J. Phys. D Appl. Phys..

[2] Berry C.C., Curtis A.S.G. (2003). Functionalisation of magnetic nanoparticles for applications in biomedicine. J. Phys. D Appl. Phys..

[3] Berry C.C. (2005). Possible exploitation of magnetic nanoparticle-cell interaction for biomedical applications. J. Mater. Chem..

[4] Mornet S., Portier J., Duguet E. (2005). A method for synthesis and functionalization of ultrasmall superparamagnetic covalent carriers based on maghemite and dextran. J. Magn. Magn. Mater..

[5] Parton E., De Palma R., Borghs G. (2007). Biomedical applications using magnetic nanoparticles. Solid State Technol..

[6] Herrera A.P., Barrera C., Rinaldi C. (2008). Synthesis and functionalization of magnetite nanoparticles with aminopropylsilane and carboxymethyldextran. J. Mater. Chem..

[7] Varadan V.K., Chen L., Xie J. (2008). Nanomedicine: Design and Applications of Magnetic Nanomaterials, Nanosensors and Nanosystems.

[8] Lascialfari A., Sangregorio C. (2011). Magnetic Nanoparticles in Biomedicine: Recent Advances. Chimica Oggi.

[9] Kim D.H., Nikles D.E., Johnson D.T., Brazel C.S. (2008). Heat Generation of Aqueously Dispersed CoFe2O4 Nanoparticles as Heating Agents for Magnetically Activated Drug Delivery and Hyperthermia. J. Magn. Magn. Mater..

[10] Chertok B., Moffat B.A., David A.E., Yu F., Bergemann C., Ross B.D., Yang V.C. (2008). Iron Oxide Nanoparticles as a Drug Delivery Vehicle for MRI Monitored Magnetic Targeting of Brain Tumors. Biomaterials.

[11] Jain T.K., Richey J., Strand M., Leslie-Pelecky D.L., Flask C.A., Labhasetwar V. (2008). Magnetic Nanoparticles with Dual Functional Properties: Drug Delivery and Magnetic Resonance Imaging. Biomaterials.

[12] Dulinska-Litewka J., Łazarczyk A., Hałubiec P., Szafranski O., Karnas K., Karewicz A. (2019). Superparamagnetic Iron Oxide Nanoparticles—Current and Prospective Medical Applications. Materials.

[13] Ulman A. (1996). Formation and Structure of Self-Assembled Monolayers. Chem. Rev..

[14] Stöber W., Fink A., Bohn E. (1968). Controlled Growth of Monodispersed Silica Spheres in the Micron Size Range. J. Colloid Interf. Sci..

[15] Jamshaid T., Zine N., Errachid El-Salhi A., Ahmad N.M., Elaissari A. (2014). Soft Nanoparticles for Biomedical Applications.

[16] Tangchaikeeree T., Polpanich D., Bentaher A., Baraket A., Errachid A., Agusti G., Elaissari A., Jangpatarapongsa K. (2017). Combination of PCR and dual nanoparticles for detection of Plasmodium falciparum. Colloids Surf. B..

[17] Jamshaid T., Eissa M.M., Lelong Q., Bonhommé A., Augsti G., Zine N., Errachid A., Elaissari A. (2017). Tailoring of carboxyl-decorated magnetic latex particles using seeded emulsion polymerization. Polym. Adv. Technol..

[18] Bray M., Yang Y., Fare C., Chai C., Jessieville F., Barakat A., Arachnid A., Zhang A.D., Jaffrezic-Renault A. (2017). Boron-doped Diamond Electrodes Modified with Fe3O4@Au Magnetic Nanocomposites as Sensitive Platform for Detection of a Cancer Biomarker, Interleukin-8. Electroanalysis.

[19] Jamshaid T., Neto E.T.T., Elissa M.M., Zine N., Hiroiuqui Kunita M., Errachid A., Elaissari A. (2016). Magnetic particles: From preparation to lab-on-a-chip, biosensors, microsystems and microfluidics applications. TrAC-Trend Anal. Chem..

[20] Amstad E., Textor M., Reimhult E. (2011). Stabilization and functionalization of iron oxide nanoparticles for biomedical applications. Nanoscale.

[21] Berry C.C. (2009). Functionalization of magnetic nanoparticles for applications in biomedicine. J. Phys. D Appl. Phys..

[22] Faraji M., Yamini Y., Rezaee M. (2010). Magnetic nanoparticles: Synthesis, stabilization, functionalization, characterization and applications. JICS.

[23] Hao R., Xing R., Xu Z., Hou Y., Gao S., Sun S. (2010). Synthesis, functionalization, and biomedical applications of multifunctional magnetic nanoparticles. Adv. Mater..

[24] Eissa M.M., Rahman M.M., Zine N., Jaffrezic-Renault N., Errachid A., Fessi H., Elaissari A. (2013). Reactive magnetic poly(divinylbenzene-co-glycidyl methacrylate) colloidal particles for specific antigen detection using microcontact printing technique. Acta Biomater..

[25] Lu A.H., Salabas E.L., Schüth F. (2007). Magnetic nanoparticles: Synthesis, protection, functionalization and applications. Angew. Chem. Int. Ed..

[26] Xu C., Sun S. (2007). Monodisperse magnetic nanoparticles for biomedical applications. Polym. Int..

[27] Gan N., Yang X., Xie D., Wu Y., Wen W. (2010). A disposable organophosphorus pesticides enzyme biosensor based on magnetic composite nano-particles modified screen printed carbon electrode. Sensors.

[28] Justino C.I.L., Rocha-Santos T.A.P., Cardoso S., Duarte A.C. (2013). Strategies for enhancing the analytical performance of nanomaterial-based sensors. Trends Anal. Chem..

[29] Justino C.I.L., Rocha-Santos T.A., Duarte A.C. (2010). Review of analytical figures of merit of sensors and biosensors in clinical applications. Trends Anal. Chem..

[30] Li J., Gao H., Chen Z., Wei X., Yang C.F. (2010). An electrochemical immunosensor for carcinoembryonic antigen enhanced by self-assembled nanogold coatings on magnetic nanoparticles. Anal. Chim. Acta.

[31] Xin Y., Fu-Bing X., Hong-Wei L., Feng W., Di-Zhao C., Zhao-Yang W. (2013). A novel H_2_O_2_ biosensor based on Fe3O4-Au magnetic nanoparticles coated horseradish peroxidase and graphene sheets-Nafion film modified screen-printed carbon electrode. Electrochim. Acta.

[32] Chen D., Deng J., Liang J., Xie J., Hue C., Huang K. (2013). A core-shell molecularly imprinted polymer grafted onto a magnetic glassy carbon electrode as a selective sensor for the determination of metronidazole. Sens. Actuators B Chem..

[33] Prakash A., Chandra S., Bahadur D. (2012). Structural, magnetic, and textural properties of iron oxide-reduced graphene oxide hybrids and their use for the electrochemical detection of chromium. Carbon.

[34] Hu Y., Zang Z., Zhang H., Luo L., Yao S. (2012). Selective and sensitive molecularly imprinted sol-gel film-based electrochemical sensor combining mercaptoacetic acid-modified PbS nanoparticles with Fe_3_O_4_@Au-multi-walled carbon nanotubes-chitosan. J. Solid State Electrochem..

[35] Arvand M., Hassannezhad M. (2014). Magnetic core–shell Fe3O4@SiO2/MWCNT nanocomposite modified carbon paste electrode for amplified electrochemical sensing of uric acid. Mater. Sci. Eng. C.

[36] Chen X., Zhu J., Chen Z., Xu C., Wang Y., Yao C. (2011). A novel bienzyme glucose biosensor based on three-layer Au-Fe_3_O_4_@SiO_2_ magnetic nanocomposite. Sens. Actuators B Chem..

[37] Halima H.B., Zine N., Gallardo-González J., Aissari A.E., Sigaud M., Alcacer A., Bausells J., Errachid A. A Novel Cortisol Biosensor Based on the Capacitive Structure of Hafnium Oxide: Application for Heart Failure Monitoring. Proceedings of the 2019 20th International Conference on Solid-State Sensors, Actuators and Microsystems & Eurosensors XXXIII (TRANSDUCERS & EUROSENSORS XXXIII).

[38] Drobitch R.K., Svensson C.K. (1992). Therapeutic drug monitoring in saliva. Clin. Pharm..

[39] Jasim H., Olausson P., Hedenberg-Magnusson B., Ernberg M., Ghafouri B. (2016). The proteomic profile of whole and glandular saliva in healthy pain-free subjects. Sci. Rep..

[40] Xu R., Cui B., Duan X., Zhang P., Zhou X., Yuan Q. (2020). Saliva: Potential diagnostic value and transmission of 2019-nCoV. Int. J. Oral. Sci..

[41] Fakheran O., Dehghannejad M., Khademi A. (2020). Saliva as a diagnostic specimen for detection of SARS-CoV-2 in suspected patients: A scoping review. Infect. Dis. Poverty.

[42] Azzi L., Carcano G., Gianfagna F., Grossi P., Gasperina D.D., Genoni A., Fasano M., Sessa F., Tettamanti L., Carinci F. (2020). Saliva is a reliable tool to detect SARS-CoV-2. J. Infect..

[43] Segal A., Wong D.T. (2008). Salivary diagnostics: Enhancing disease detection and making medicine better. Eur. J. Dent. Educ..

[44] Lee Y.H., Wong D.T. (2009). Saliva: An emerging biofluid for early detection of diseases. Am. J. Dent..

[45] Javaid M.A., Ahmed A.S., Durand R., Tran S.D. (2016). Saliva as a diagnostic tool for oral and systemic diseases. J. Oral Biol. Craniofac. Res..

[46] Golas P.L., Louie S., Lowry G.V., Matyjaszewski K., Tilton R.D. (2010). Comparative study of polymeric stabilizers for magnetite nanoparticles using ATRP. Langmuir.

[47] Yu W., Xie H. (2012). A review on nanofluids: Preparation, stability mechanisms, and applications. J. Nanomater..

[48] Matlochová A., Plachá D., Rapantová N. (2013). The application of nanoscale materials in groundwater remediation. PJoES.

[49] Svoboda J. (2005). Encyclopedia of Materials: Science and Technology.

[50] Hassani H., Zakerinasab B., Nasseri M.A., Shavakandi M. (2016). The preparation, characterization and application of COOH grafting on ferrite-silica nanoparticles. Rsc Adv..

[51] Kralj S., Drofenik M., Makovec D. (2010). Controlled surface functionalization of silica-coated magnetic nanoparticles with terminal amino and carboxyl groups. J. Nanopart. Res..

[52] O’Handley R.C. (1999). Modern Magnetic Materials: Principles and Applications.

