# Supplementary material for: Magnetic Nanoparticles Fishing for Biomarkers in Artificial Saliva
Source: Molecules. 2020 Aug 31;25(17):3968. doi: 10.3390/molecules25173968 (PMC7504804; doi:10.3390/molecules25173968)
Supplement: Supplementary file 1 [file molecules-25-03968-s001.pdf]

Magnetic Nanoparticles Fishing for Biomarkers in Artificial Saliva

~~Magnetic Nanoparticles Fishing Antigens in Saliva~~

Arpita Saha[1], Hamdi Ben Halima[2], Abhishek Saini[1], Juan Gallardo-Gonzalez[2], Nadia zine[2], Clara Vinas[1], Abdelhamid El Aissari[3], Abdelhamid Errachid[2] and Francesc -Teixidor[1]

[1]Institut de Ciencia de Materials de Barcelona (ICMAB-CSIC), Campus de la UAB, 08193, Bellaterra, Spain
[2]Université de Lyon, Institut des Science Analytiques, UMR 5280, CNRS, Université Lyon 1, ENS Lyon -5, rue de la Doua, F-69100 Villeurbanne, France
[3]Université de Lyon, LAGEP, UMR-5007, CNRS, Université Lyon 1, 5007, 43 Bd 11 Novembre 1918, F-69622 Villeurbanne, France

Table of Contents

Table of Contents 1
Results and Discussion Images..1

Results and Discussion Images

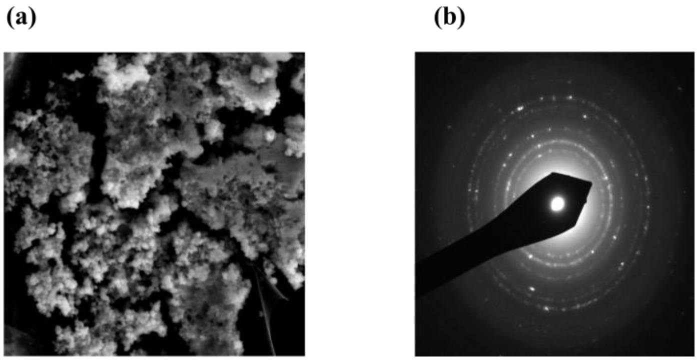

Figure S1. (a) The SEM of Fe_3O_4@SiO_2-COOH MNPs and (b) the electron diffraction pattern.

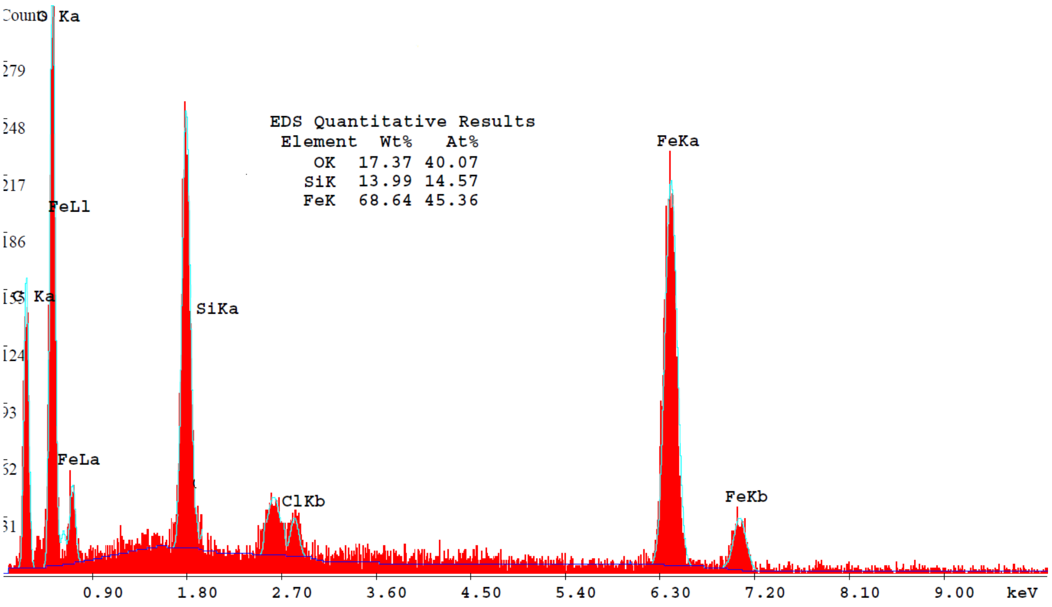

Figure S2. The EDS of $Fe_3O_4@SiO_2$-COOH MNPs shows Fe, O and Si.

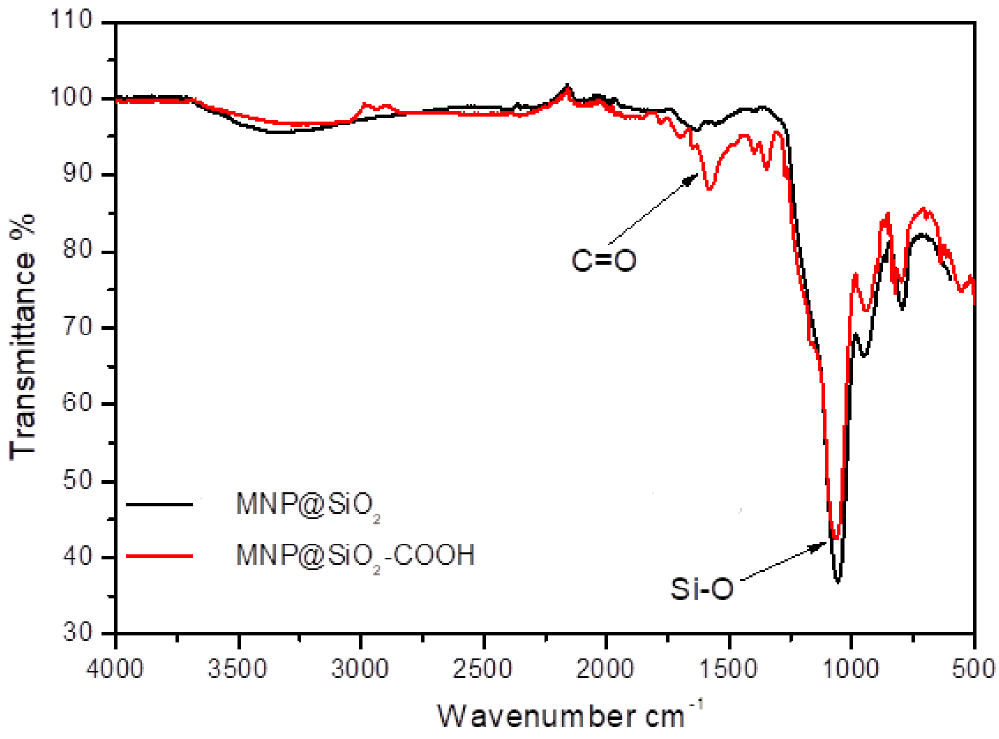

Figure S3. The IR spectrum of $Fe_3O_4@SiO_2$-COOH MNPs and $Fe_3O_4@SiO_2$. The peak of Si-O and C=O is visible in the respective spectra.

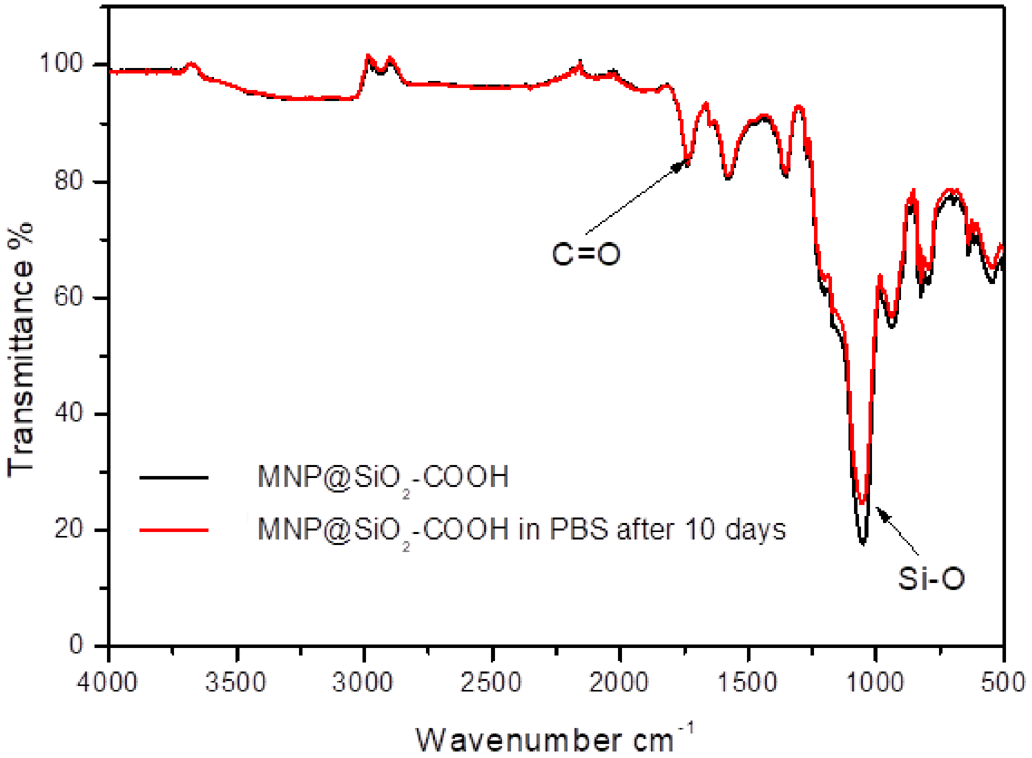

Figure S4. The IR spectrum of $Fe_3O_4@SiO_2$-COOH MNPs before and after being dispersed in PBS for 10 days was tested. It maintained its functionalization.

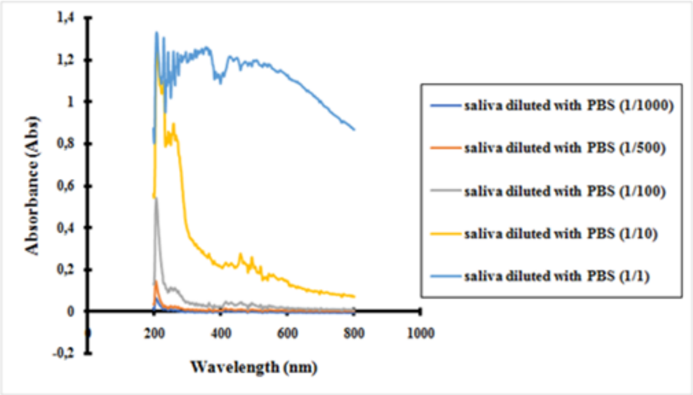

Figure S5. UV-vis spectra of saliva at different dilution ratios saliva/PBS.

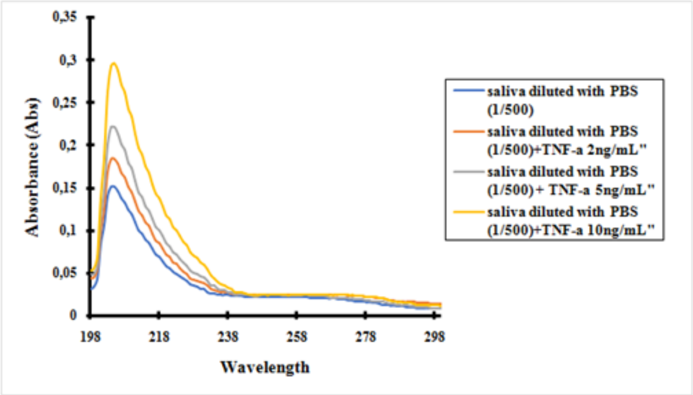

Figure S6. UV-vis spectra of TNF-α at different concentrations in a fixed background of artificial saliva diluted at 1/500 with PBS.

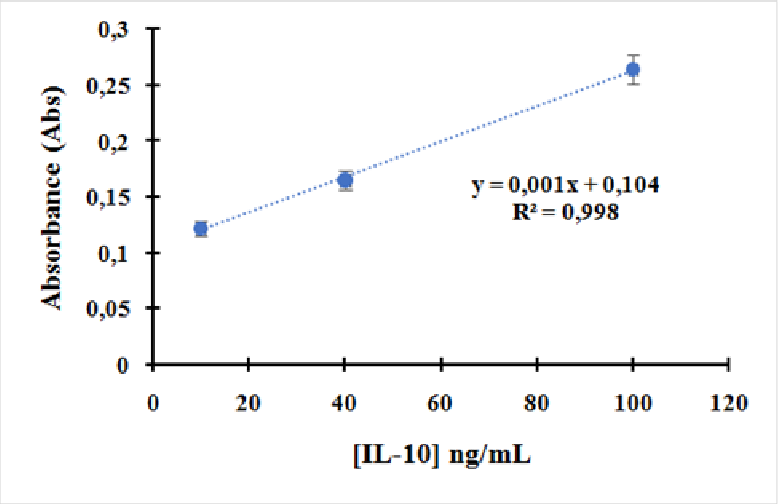

Figure S7. Calibration curve of IL-10 at different concentrations: 10, 40 and 100 ng/mL. Fixed background of artificial saliva diluted 1/500 with 10 mM PBS. Error bars correspond to three replicates per sample.

A new complex MNP@SiO_2-NH_2-CO-anti-IL-10 was prepared using the experimental procedure described before and using a fixed concentration of anti-IL antibody of 10 ng/mL for the bio-functionalization of MNPs. Subsequently, IL-10 was incubated with the complex MNP@SiO_2-NH_2-CO-anti-IL-10 at different concentrations: 10, 40 and 100 ng/mL. The supernatants containing the unreacted IL-10 were measured and compared to a reference previously prepared (Fig. 12).

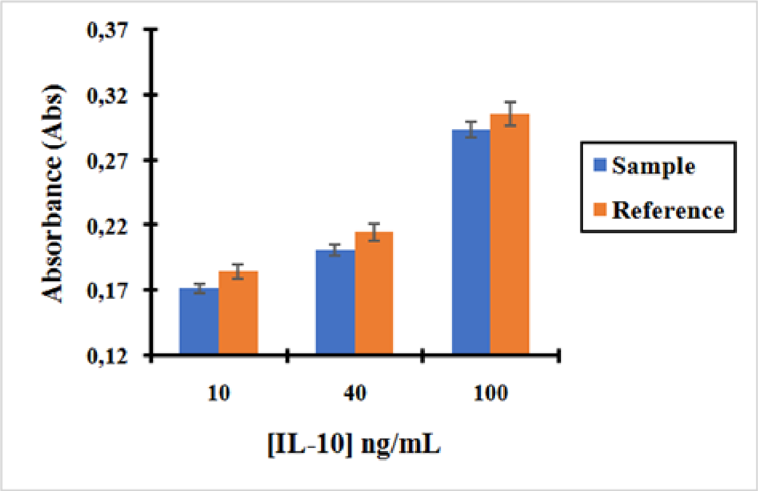

Figure S8. Bar graph showing (blue) the measurement of the absorbance after IL-10 incubation with the complex MNP@SiO_2-NH_2-CO-anti-IL-10 at three different concentrations of IL-10: 10, 40 and 100 ng/L; (Orange) the measurement of the absorbance of IL-10 after incubation with none activated MNPs. Background fixed in artificial saliva at 1/500. Error bars correspond to three replicates per sample.

Mechanism:Activation of the -COOH groups with EDC/NHS of Fe_3O_4@SiO_2-NH_2-COOH

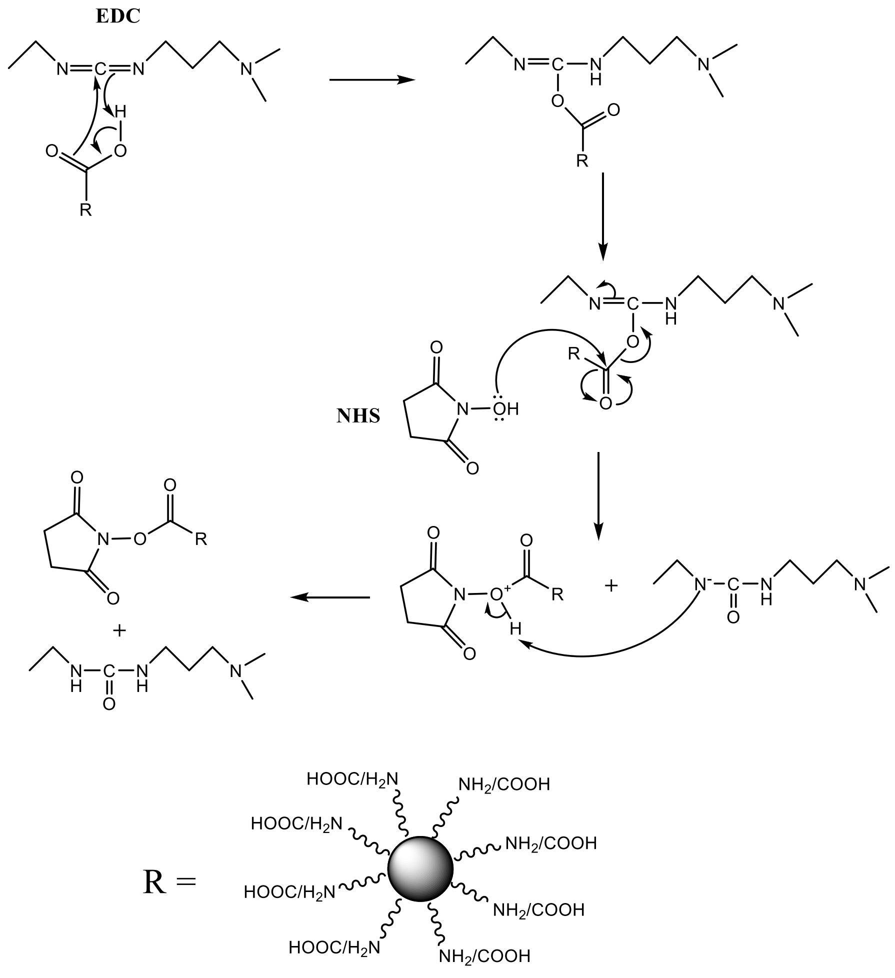

Fe_3O_4@SiO_2-NH_2-COOH

Figure S9. Reaction mechanism corresponding to the activation of carboxylic acids using the mixture EDC/NHS.

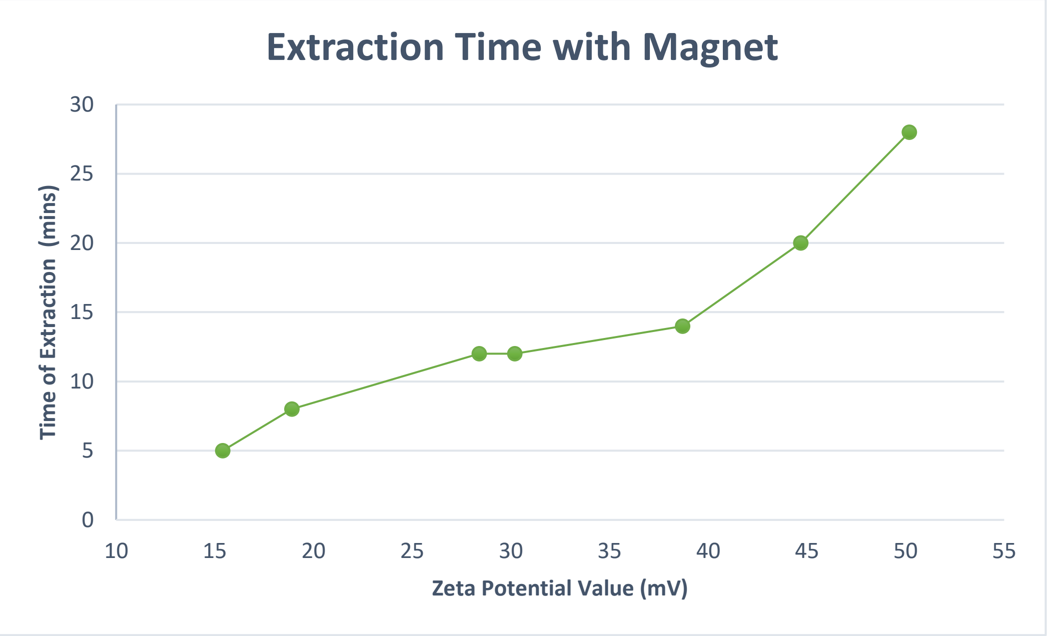

Figure S10: Time of extraction of MNPs with increasing zeta-potential using Dimethyl di-octadecyl ammonium chloride.

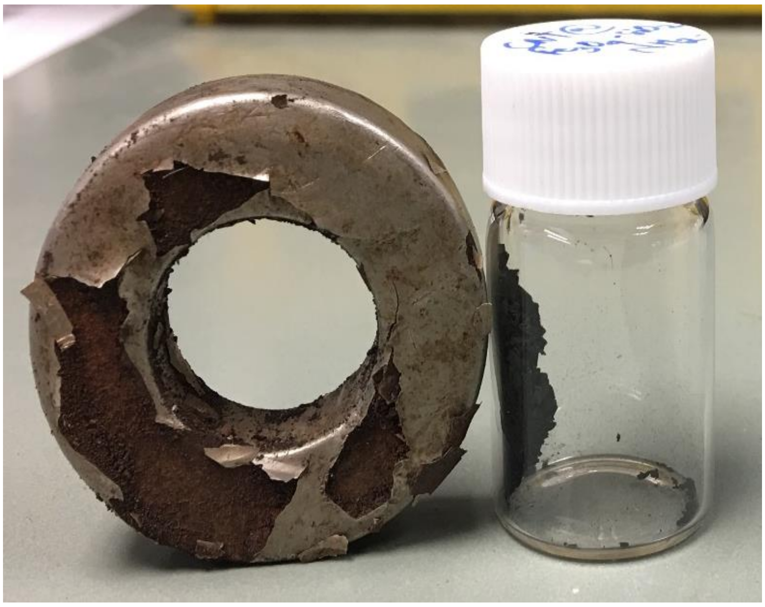

Figure S11: Photograph of MNPs being extracted by an external magnet.

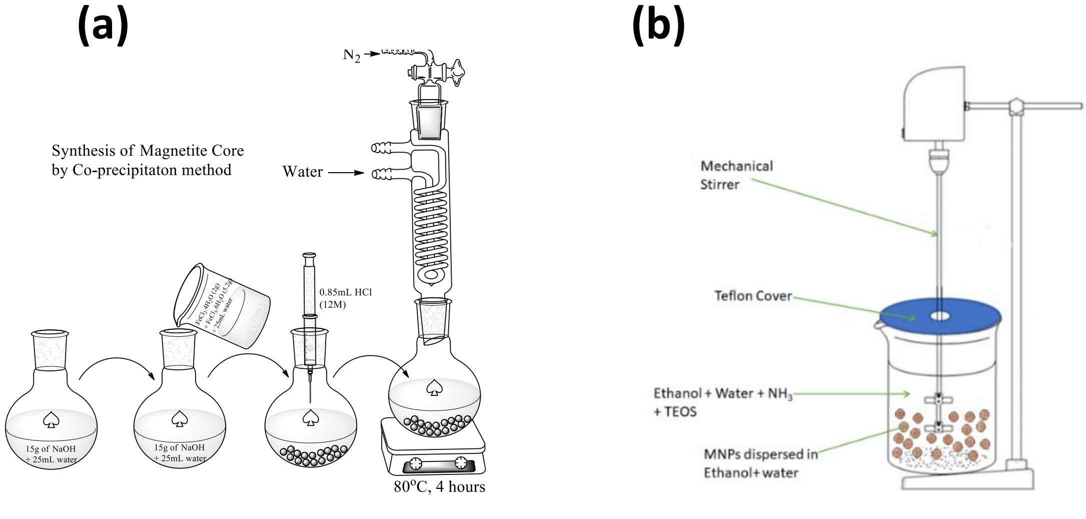

Figure S12: Schematic of synthesis of (a) Fe_3O_4 core by co-precipitation method and (b) SiO_2 shell by Stober method.

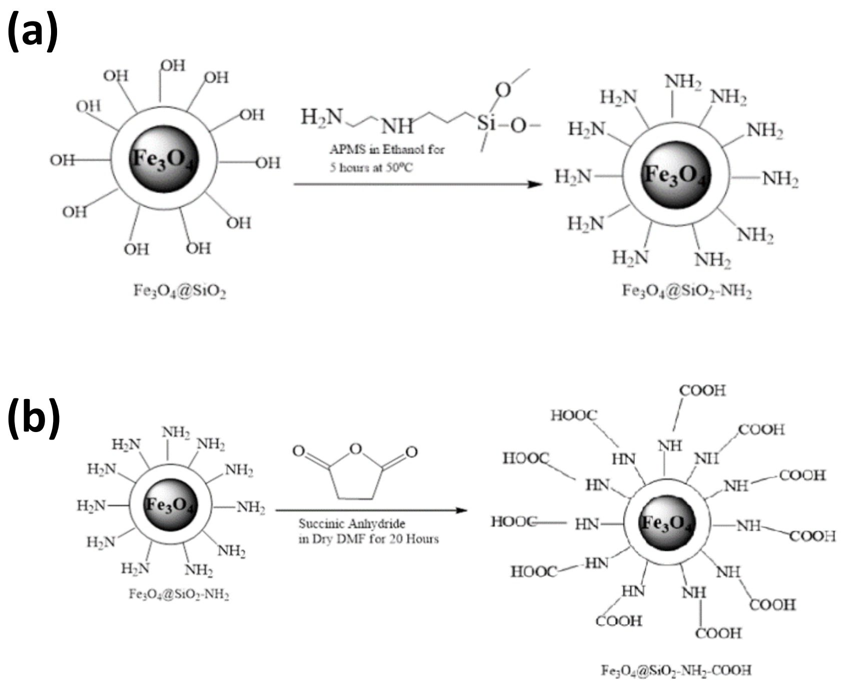

Figure S13: Synthesis of (a) $Fe_3O_4@SiO_2-NH_2$ and then (b) $Fe_3O_4@SiO_2-NH_2-COOH$.

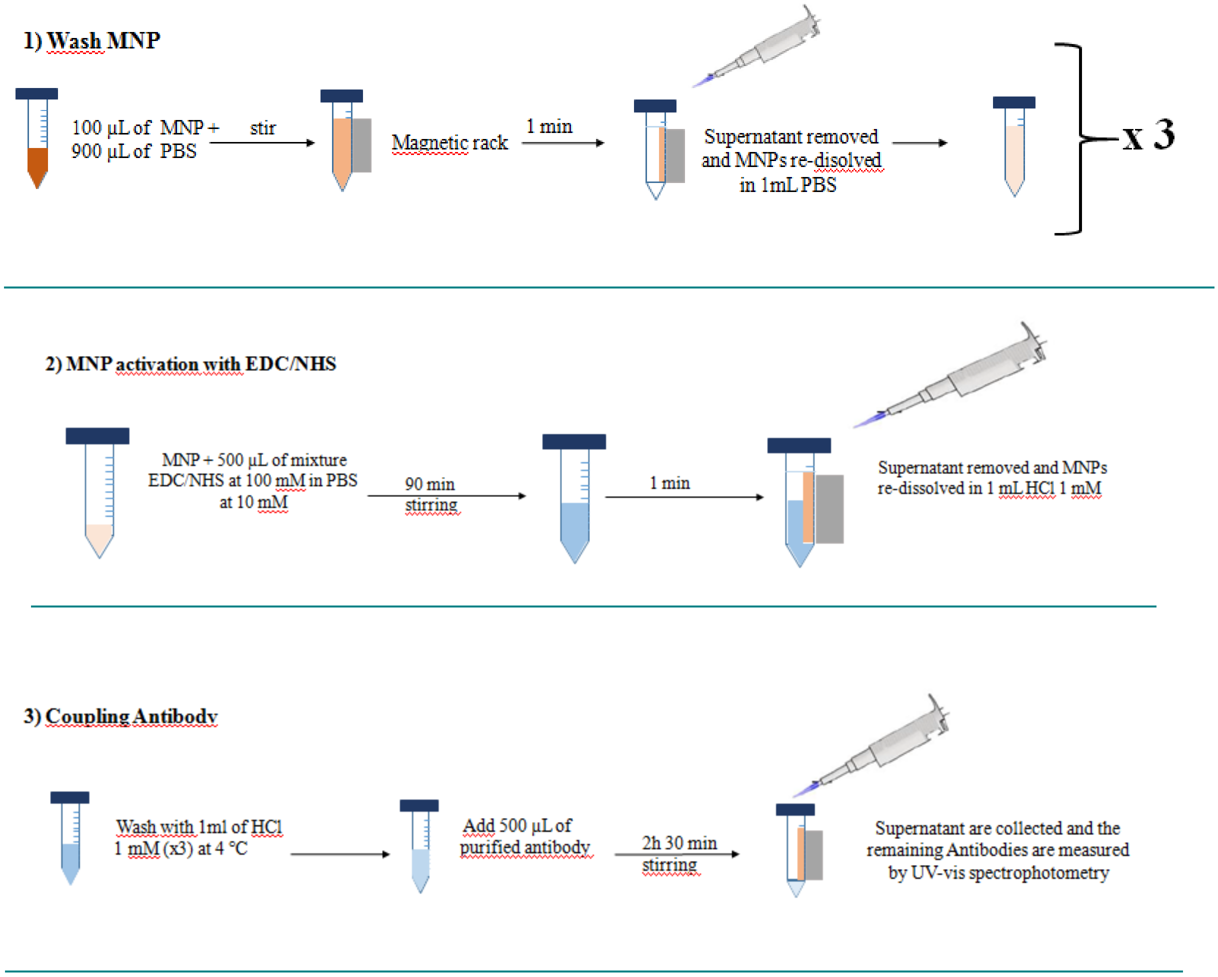

Figure S14: Schematic illustration of the experimental procedure for the bio-functionalization of MNPs with antibodies.

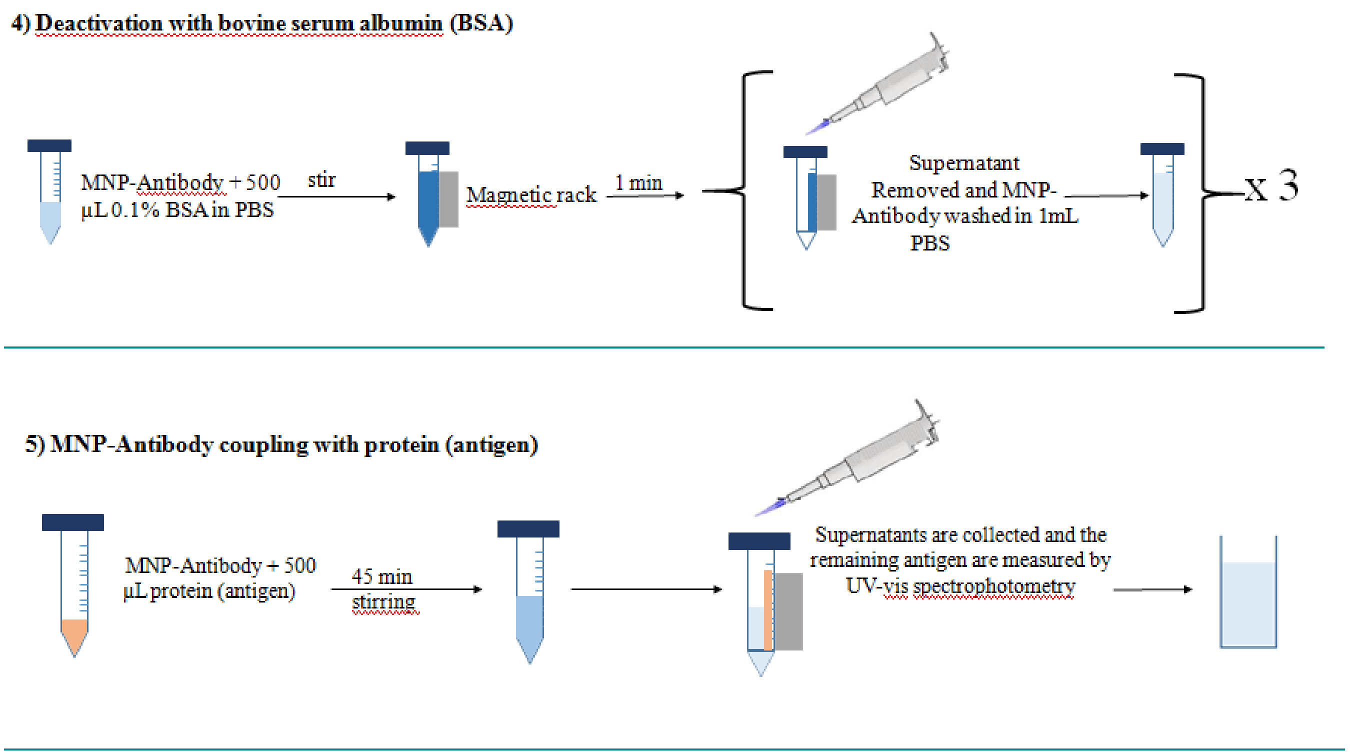

Figure S15: Schematic illustration of the experimental procedure for the pre-concentration of TNF-α using the complex MNP@SiO_2-NH_2-CO-anti-TNF-α.

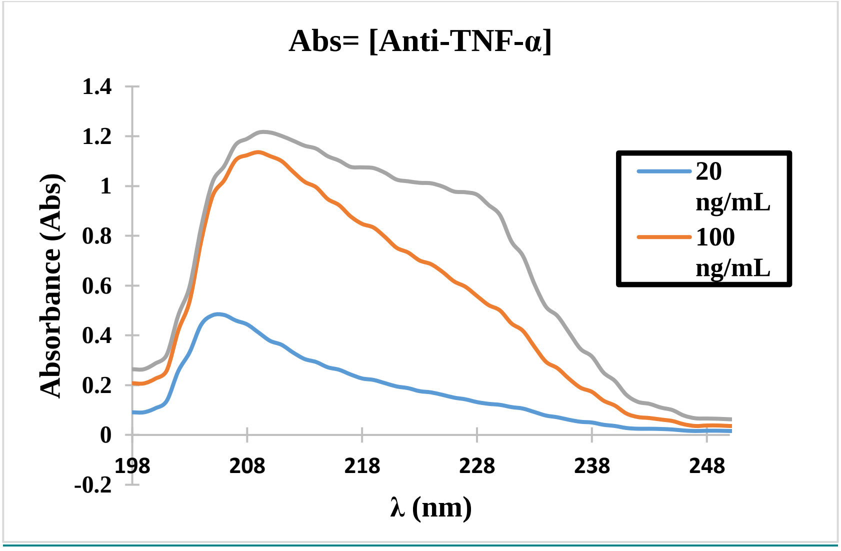

Figure S16: UV-vis spectrum of Anti-TNF-α antibody in PBS at different concentrations: 20, 100 and 200 ng/mL.

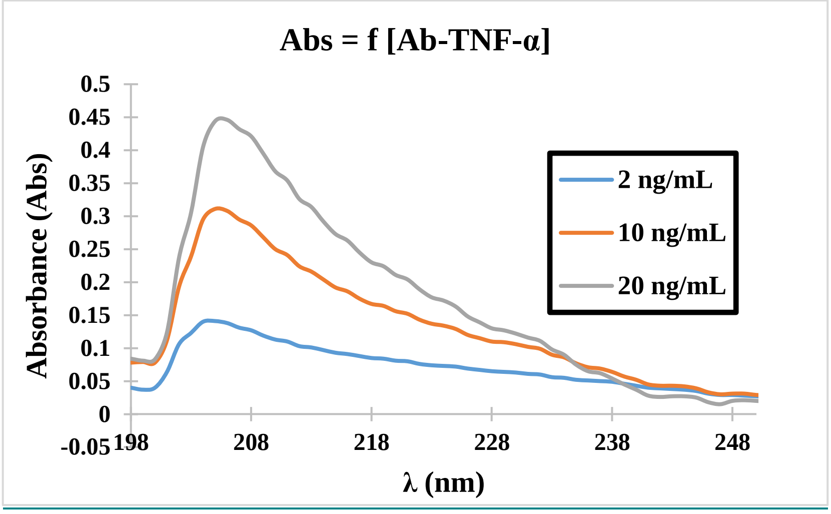

Figure S17: Example of UV-vis spectrum of Anti-TNF-*α* antibody in PBS at different concentrations: 2, 10 and 20 ng/mL.

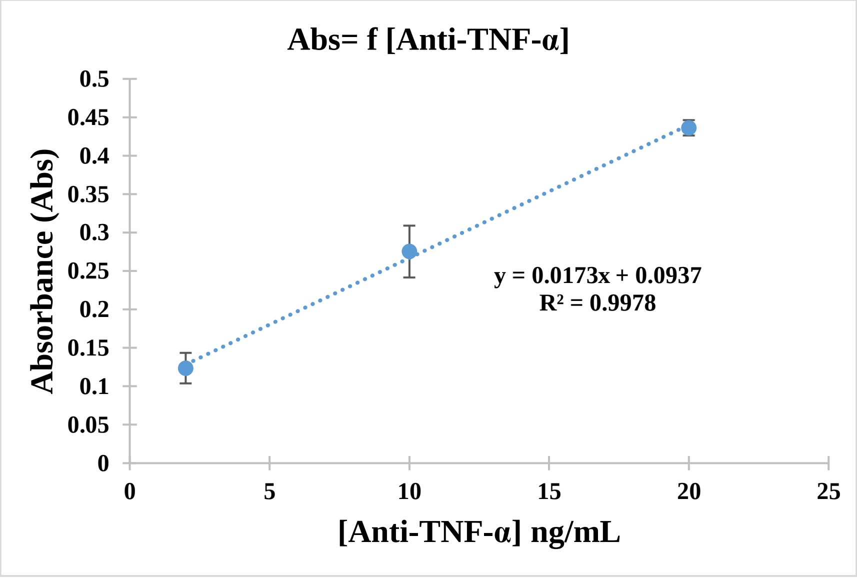

Figure S18: Calibration curve of anti-TNF-*α* antibody at different concentrations: 2, 5 and 10 ng/mL in PBS. Error bars correspond to three replicates per sample.

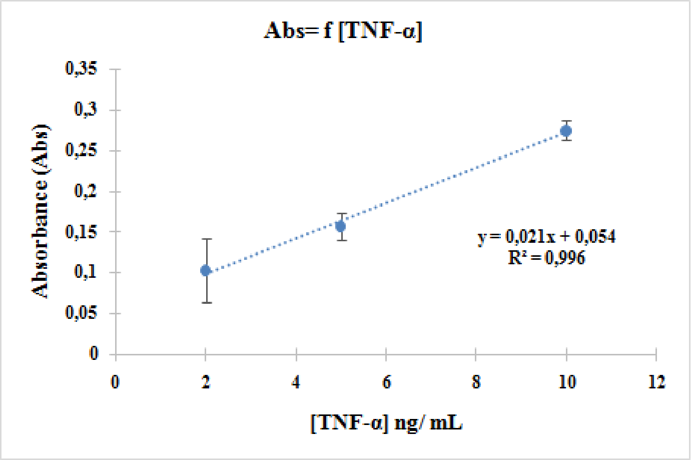

Figure S19: Calibration curve of TNF-*α* at different concentrations: 2, 5 and 10 ng/mL in PBS. Error bars correspond to three replicates per sample.
